# A Microvascularized Tumor-mimetic Platform for Assessing Anti-cancer Drug Efficacy

**DOI:** 10.1038/s41598-018-21075-9

**Published:** 2018-02-16

**Authors:** Shantanu Pradhan, Ashley M. Smith, Charles J. Garson, Iman Hassani, Wen J. Seeto, Kapil Pant, Robert D. Arnold, Balabhaskar Prabhakarpandian, Elizabeth A. Lipke

**Affiliations:** 1 Department of Chemical Engineering, Auburn University, Auburn, AL 36849 USA; 2 Biomedical Technology, CFD Research Corporation, Huntsville, AL 35806 USA; 3 Department of Drug Discovery and Development, Auburn University, Auburn, AL 36849 USA

## Abstract

Assessment of anti-cancer drug efficacy in *in vitro* three-dimensional (3D) bioengineered cancer models provides important contextual and relevant information towards pre-clinical translation of potential drug candidates. However, currently established models fail to sufficiently recapitulate complex tumor heterogeneity. Here we present a chip-based tumor-mimetic platform incorporating a 3D *in vitro* breast cancer model with a tumor-mimetic microvascular network, replicating the pathophysiological architecture of native vascularized breast tumors. The microfluidic platform facilitated formation of mature, lumenized and flow-aligned endothelium under physiological flow recapitulating both high and low perfused tumor regions. Metastatic and non-metastatic breast cancer cells were maintained in long-term 3D co-culture with stromal fibroblasts in a poly(ethylene glycol)-fibrinogen hydrogel matrix within adjoining tissue chambers. The interstitial space between the chambers and endothelium contained pores to mimic the “leaky” vasculature found *in vivo* and facilitate cancer cell-endothelial cell communication. Microvascular pattern-dependent flow variations induced concentration gradients within the 3D tumor mass, leading to morphological tumor heterogeneity. Anti-cancer drugs displayed cell type- and flow pattern-dependent effects on cancer cell viability, viable tumor area and associated endothelial cytotoxicity. Overall, the developed microfluidic tumor-mimetic platform facilitates investigation of cancer-stromal-endothelial interactions and highlights the role of a fluidic, tumor-mimetic vascular network on anti-cancer drug delivery and efficacy for improved translation towards pre-clinical studies.

## Introduction

Cancer cell invasion, migration, intravasation and extravasation are key events, amongst others, in driving the complex phenomena of tumor malignancy and metastasis^1,2^. The synergistic interplay between cancer cells and surrounding stromal components (including cancer-associated fibroblasts, endothelial cells, and extracellular matrix (ECM) proteins) influences the overall course of disease progression and response to anti-cancer therapeutics^2,3^. Recapitulation of the complex and heterogeneous tumor microenvironment (TME) with a high degree of physiological relevancy in *in vitro* systems is a significant challenge, which has led to the development of several biomimetic three-dimensional (3D) models that can capture key aspects of the tumor milieu for investigations in cancer research^4–6^. Recent advances in biofabrication techniques have enabled the use of organ-on-a-chip systems for recapitulating the complexities of the human physiology^7–9^; these micro-scale platforms significantly reduce cost, labor and time compared to *in vivo* models while still providing important, contextual information for further translation in pre-clinical studies. In this context, microfluidic cancer-on-a-chip platforms have also emerged as a valuable tool for the investigation of malignant and metastatic processes in the TME and for assessment of efficacies of anti-cancer therapeutics^10–15^.

Bioengineered 3D cancer models developed till date incorporate varying degrees of pathological complexity with respect to that found in native tumors. The incorporation of stromal fibroblasts and supporting cell types within ECM-mimic matrices and scaffolds lends additional physiological context to these cancer models^4,6^. Co-culture of stromal fibroblasts and supporting cell types with cancer cells in 3D microenvironments allow for investigation of vital intercellular interactions and bidirectional signaling mechanisms involved in tumor progression and malignancy^4,6^. In addition, the presence of specific topographical, physical, mechanical and biochemical cues in the stromal ECM also influence 3D malignant behavior^16,17^. However, the majority of cancer-on-a-chip platforms are highly reductionist and comparatively simplistic in relation to native, vascularized tumors and designed to study specific events of tumor progression (including extravasation, angiogenesis, bidirectional cell-cell signaling) rather than facilitate holistic interrogation of cancer as an ‘organ’ with its surrounding interactive microenvironment^15,18^. Although it is known that uniform delivery of chemotherapeutics in native tumors is impeded by the disorganized, leaky and abnormal tumor vasculature, microfluidic systems and current *in vitro* models have yet to exploit and investigate the role of these irregular vascular features in the transport processes. In addition, the impact of on-chip tumor microvascular architecture and flow patterns on the delivery, penetration and uptake of anti-cancer therapeutics into the central tumor tissue is yet to be explored.

The use of biomaterial-based scaffolds and matrices in the development of 3D *in vitro* cancer models has facilitated the recapitulation of tumor ECM and its mutual crosstalk with cancer cells and supporting stromal cell-types^19^. Some common ECM-mimetic biomaterials include collagen, Matrigel, alginate, silk fibroin and peptide-conjugated poly(ethylene glycol) (PEG)-based hydrogels, amongst others^20,21^. In this study, we explore the use of PEG-fibrinogen (PF), a previously underutilized biomaterial in cancer studies, for investigation of 3D cancer-ECM and cancer-endothelial interactions. PF, obtained by the covalent coupling of poly(ethylene glycol diacrylate) (PEGDA) and fibrinogen, is readily photocrosslinkable in the presence of Eosin Y under visible light to yield biocompatible hydrogels and has been previously used for a number of applications including cardiogenic differentiation of human induced pluripotent stem cells (hiPSCs)^22^, chondrogenic differentiation of human bone marrow derived mesenchymal stem cells (hBM-MSCs)^23^ and investigation of cellular morphogenesis of human fibroblasts^24^. Hence, incorporation of fibrinogen in the stromal matrix of bioengineered 3D cancer models provides a unique opportunity to explore cancer-ECM interactions, 3D cancer cell behavior and responsiveness to anti-cancer drugs.

In this study, we present a microfluidic tumor-mimetic platform that closely mimics native tumor vascular flow dynamics and enables the investigation of cancer-ECM-endothelial interactions. The microfluidic network facilitated the formation of an intricate, tumor-mimetic, vascular endothelium when maintained under physiological shear flow. Cancer cells and fibroblasts were maintained in long-term co-culture (*e.g*., 4 weeks) with time-dependent changes in morphological features characteristic of the cancer cell-type. The role of the microvascular network pattern on the vascular shear flow rates and subsequent gradients of macromolecular diffusion experienced by the cancer cells was also investigated. Finally, vascular pattern-dependent anti-cancer drug efficacy of doxorubicin and paclitaxel on the cancer cells and their associated cytotoxicity on endothelial cells were also analyzed. Overall, the developed tumor-mimetic platform incorporates multiple degrees of pathophysiological complexities (3D contextual cancer-fibroblast-ECM crosstalk, vascular flow variations and concentration gradients) and facilitates investigation of cell-cell and cell-matrix interactions, bulk tumor tissue stiffness, long-term studies of cancer invasiveness and malignancy, and systematic analysis of drug efficacy and cytotoxicity on the cancer tissue as a whole ‘organ’. With further optimization, the established tumor-mimetic platform can be extended for future investigations of tumorigenic mechanisms and evaluation of delivery and efficacy and safety of other organs for the novel therapeutics.

## Results

### Design and fabrication of tumor-mimetic chips

**Figure 1:**
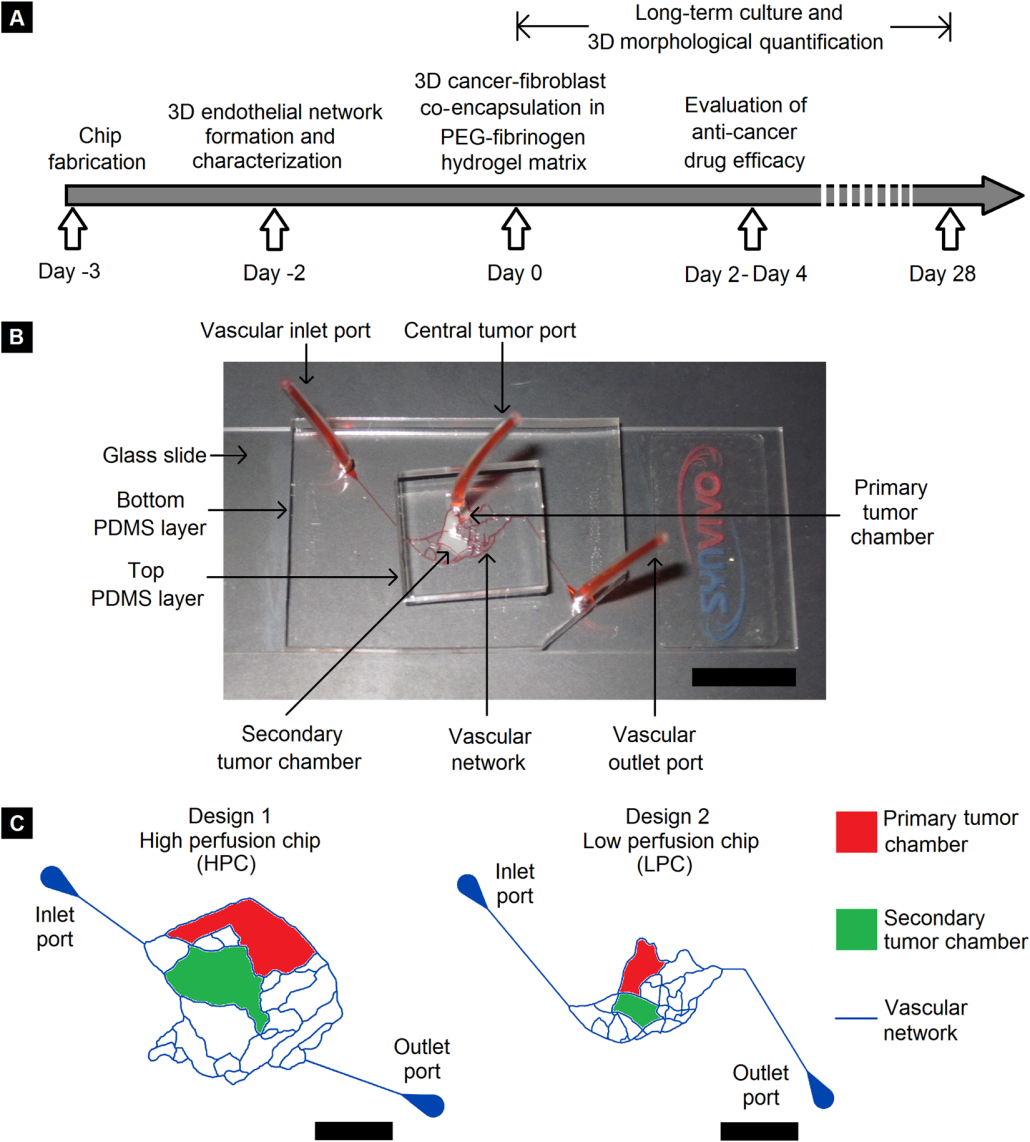
Tumor-mimetic chip design and experimental workflow. (**A**) Experimental timeline of tumor-mimetic chip fabrication, vascular network formation, 3D cancer-fibroblast encapsulation and long-term co-culture and drug testing. (**B**) Photograph of the tumor-mimetic chip perfused with Eosin Y (Scale bar = 1 cm). (**C**) Schematic representation of the vascular network, primary and secondary tumor chambers of two different designs used in the study (Scale bar = 5 mm).

The tumor-mimetic chips used in this study were fabricated using polydimethylsiloxane (PDMS)-based photolithographic techniques as described in previous studies^25,26^ and detailed in the Methods section. Two specific microvascular designs obtained from mouse vasculature were used as templates for tumor-mimetic chip fabrication; the two resulting chips will henceforth be referred to as the “high perfusion chip” (HPC) and the “low perfusion chip” (LPC). This designation is based on the comparative degree of fluidic exchange between the microvascular channels and the central tumor chamber with the HPC and LPC tumor-mimetic chip designs representing well-perfused and poorly-perfused native tumors, respectively. Each chip consists of a bottom PDMS layer bonded to a glass slide and a top PDMS layer bonded to the bottom PDMS layer (Fig. 1B). Vascular inlet and outlet ports (diameter = 1.5 mm) (on the bottom PDMS layer) facilitate the flow of media reagents and seeding of endothelial cells in the microfluidic network. The central tumor port is located on the top PDMS layer and is directly connected to the primary tumor chamber via a vertical channel of diameter 0.75 mm. The vascular channels (height = 100 μm, width = 100 μm) are separated from the primary and secondary tumor chambers (height = 100 μm) via an interstitial gap (referred to as “vascular gap”) of width 100 μm. The tumor chambers have pillars 25 μm diameter separated by 50 µm to allow additional support for tumors growing in 3D architectures. This vascular gap is fitted with PDMS pillars of diameter 20 μm and pillar-to-pillar spacing of 20 μm to (a) facilitate fluidic exchange of media components and reagents from the vascular channels to the tumor chambers, and (b) facilitate cancer-endothelial cell interactions. The tumor chambers of the HPC chip can culture larger tumors (~1.5 mm^3^) while the tumor chambers of the LPC can culture smaller tumors (~0.5 mm^3^) (Fig. 1C).

### Establishment of tumor-mimetic microvasculature

**Figure 2:**
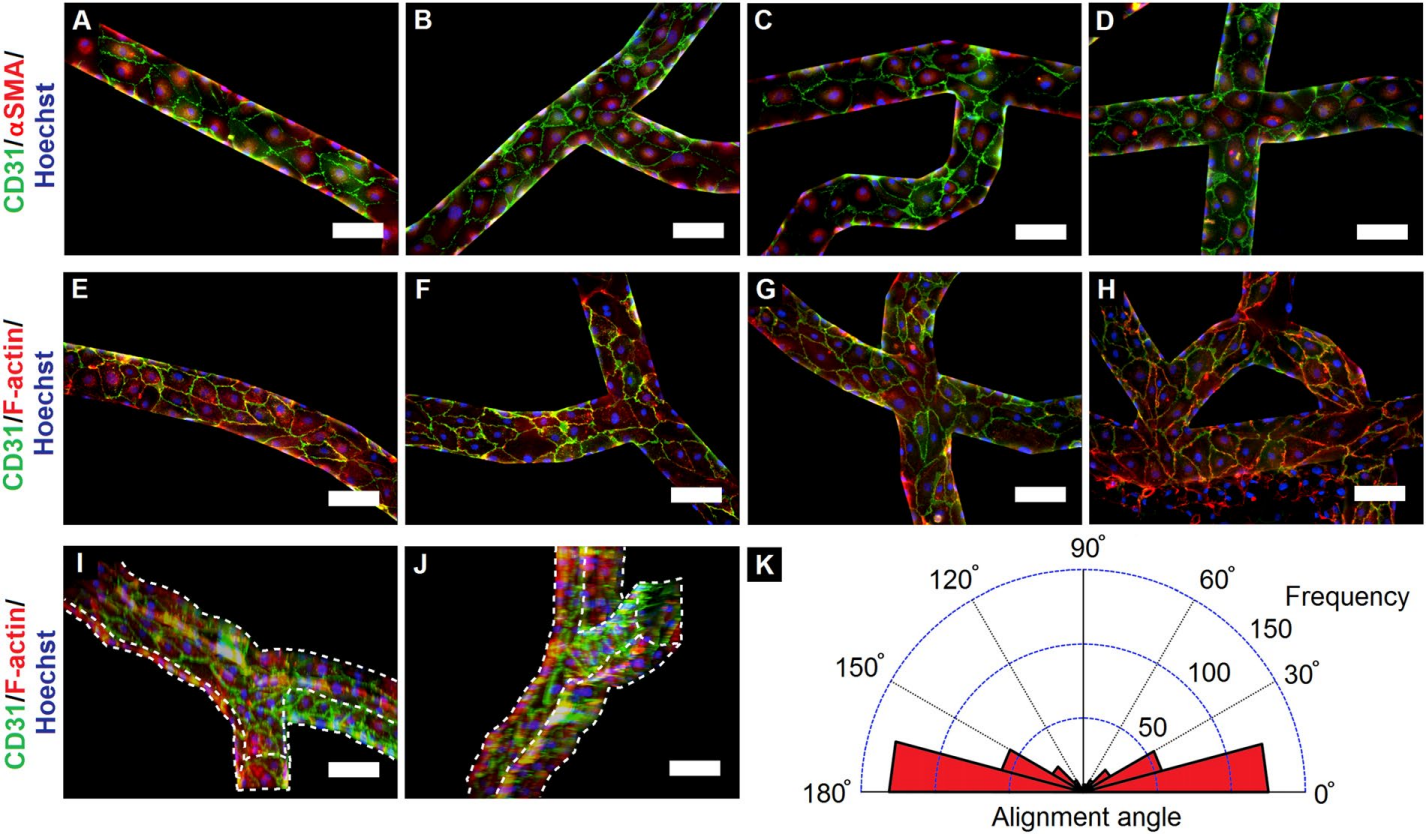
Formation and characterization of microvascular network. (**A**–**H**) Lumenized vasculature developed by human breast tumor-associated endothelial cells (hBTECs) maintained under flow in various sections of the microfluidic channels; cells displayed an elongated morphology with a high degree of vascular maturity and cell-cell contact. (**A**–**D**) (CD31, green; αSMA, red; nuclei, blue) and (**E**–**H**) hBTECs (CD31, green; F-actin, red; nuclei, blue) (**I**–**J**) 3D projected views of vascular channels exhibited complete cellular coverage of the channels (white lines indicate edges of microchannel). Scale bar = 100 μm. Vascular flow is oriented from left to right and from top to bottom in the images. (**K**) Circular histogram of hBTEC orientation when maintained under continuous flow. A high degree of cellular alignment with respect to flow direction was observed in the channels (n = 444 cells from over 10 separate locations on each of three independent chips).

In order to recapitulate the vascular structure and dynamics of native tumor tissue, a mature, lumenized microvascular network comprised of human breast tumor-associated endothelial cells (hBTECs) was first established within the tumor-mimetic chips. hBTECS were seeded within fibronectin-coated microfluidic channels and allowed to attach overnight. hBTECs maintained under continuous vascular flow for 2 days demonstrated complete lumenization and channel wall coverage throughout the microvascular network indicative of uniform cell attachment, cell spreading and proliferation (Fig. 2I,J, see Supplementary Movie S1). Immunostaining and fluorescence imaging of endothelialized channels revealed a high degree of positive expression for vascular markers CD31 and α-smooth muscle actin (αSMA) (Fig. 2A–D) in various sections of the microvascular network (including linear sections, bifurcations, X-junctions, loops and bends), indicative of a confluent endothelium and endothelial barrier function^27–29^. CD31 was prominently expressed at interendothelial cell adhesion junctions indicative of well-established cellular interconnections, which is characteristic of endothelial cells maintained under physiological shear flow. Cells stained for CD31 and actin filaments also displayed consistent, spread morphology throughout the network (Fig. 2E–H). Quantification of cellular alignment (cellular orientation relative to flow direction) revealed that the vast majority of hBTECs (~83%) aligned to within ±30° of the direction of flow; this finding was consistent across various sections of the microvasculature, indicating the prominent influence of flow in directing endothelial cell orientation (Fig. 2K, see Supplementary Fig. S1). A small fraction of cells located in junction regions experienced flow from multiple directions and hence did not show preferential alignment in a single direction. Further quantification of endothelial cell morphology was conducted from the fluorescence images in multiple sections of the vascular network with results presented in Table 1. Overall, the microfluidic chips facilitated the formation of an intricate network of mature, lumenized tumor-mimetic vasculature, which presents a significant advancement over current microfluidic systems having simplistic and idealized flow patterns.

**Table 1:** Quantification of endothelial cell morphology.

Morphological parameter	hBTECs in tumor-mimetic chips
Cell surface area (μm^2^)	2220 ± 790
Geometric diameter (μm)	53 ± 10
Cellular circularity	0.6 ± 0.1
Cellular aspect ratio	2.5 ± 0.8
Cell density (cells/mm^2^)	285 ± 24
Elongation length (μm)	92 ± 21

Morphometric characteristics of human breast tumor associated endothelial cells (hBTECs) seeded within tumor-mimetic chips and maintained under physiological flow for 48 hours. Cellular characteristics were measured from fluorescence images presented in Fig. 2. Data presented as mean ± standard deviation (n = 444 cells from three independent chips).

### Establishment of PF hydrogel-based 3D cancer-fibroblast co-culture within tumor-mimetic chips

**Figure 3:**
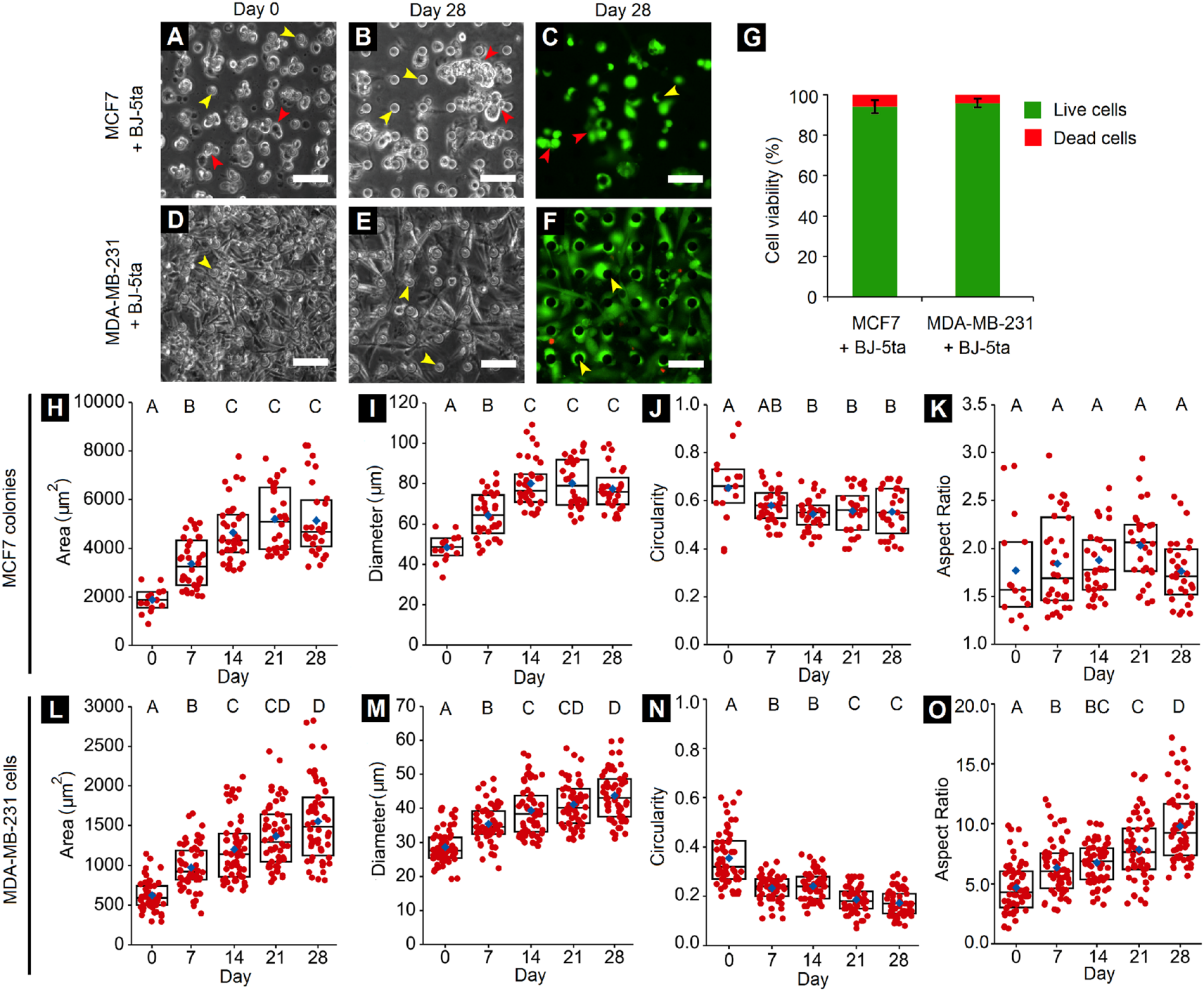
Cancer-fibroblast long-term 3D co-culture and morphological quantification. (**A**,**B**) MCF7 breast cancer cells and BJ-5ta fibroblasts co-encapsulated in PEG-fibrinogen hydrogel matrix within tumor-mimetic chips and maintained in culture for 28 days. (**C**) Live/Dead staining of cells (calcein AM, green; ethidium homodimer, red) demonstrates cell viability within chips (Red arrowheads indicate MCF7 colonies). (**D,E**) MDA-MB-231 breast cancer cells and BJ-5ta fibroblasts maintained in 3D culture within chips and (**F**) Stained for viability in a similar manner as MCF7 cells (yellow arrowheads indicate PDMS pillars of height 100 μm that support the 3D tumor chamber; Scale bar = 50 μm). (**G**) Quantification of viability within chips on day 28 of culture revealed high long-term cell viability both cancer cell types (n = 6 representative images from 3 independent chips per condition, error bars represent standard deviation). (**H**–**K**) Quantification of MCF7 colony morphology indicated increasing colony size and colony spreading over 28 days. (**L–O**) Quantification of MDA-MB-231 cell morphology exhibited increased cell spreading and elongation over 28 days. Red points denote individual MCF7 colonies/MDA-MB-231 cells and blue diamonds represent mean of respective groups. Rectangular boxes represent upper quartile, median and lower quartile of respective group. Groups having different letters have significantly different means (p < 0.05); n = minimum 15 MCF7 colonies and minimum 50 MDA-MB-231 cells from 3 independent chips per time point.

Following the formation of lumenized microvasculature within the tumor-mimetic chips, the central tumor mass, comprised of cancer cells, fibroblasts and ECM-mimetic PF hydrogel matrix, was established for long-term 3D co-culture within the tumor-mimetic chips. Either non-metastatic MCF7 or metastatic MDA-MB-231 breast cancer cells (cell seeding density: 50 × 10^6^ cells/ml) were resuspended with immortalized human foreskin BJ-5ta fibroblasts (cell seeding density: 10 × 10^6^ cells/ml) (cancer cell:fibroblast ratio = 5:1) within PF hydrogel precursor and seeded within the primary tumor chamber of vascularized chips (volume = 4–5 μl). This cell-laden hydrogel precursor was then photocrosslinked under visible light to obtain a cell-encapsulated hydrogel matrix, representative of a central tumor-stromal-ECM mass surrounded by tumor-mimetic microvasculature. Both MCF7 and MDA-MB-231 cells were distributed uniformly throughout the central tumor chamber on day 0 (Fig. 3A,D). MCF7 cells gradually formed distinct local colonies with tight cellular packing, over 28 days in culture, reminiscent of their epithelial phenotype (Fig. 3B). MDA-MB-231 cells assumed elongated and invasive morphologies as is characteristic of their native metastatic nature (Fig. 3E). Live/Dead staining of the cultured cells revealed high cell viability (>90%) for both cancer cell types within the tumor-mimetic chips at the end of 28 days (Fig. 3C,F,G).

Quantification of cellular morphological features was conducted from phase contrast images every 7 days post-encapsulation of cancer cells. For MCF7 cells (co-encapsulated with fibroblasts), there was an increase in colony area (~1,900 μm^2^ on day 0 vs. ~4,200 μm^2^ on day 28), an increase in colony diameter (~50 μm on day 0 vs. ~80 μm on day 28), and a decrease in colony circularity (~0.7 on day 0 vs. ~0.6 on day 28), but no significant change in colony aspect ratio (~1.5–2.0) over time (Fig. 3H–K). Increased colony area and diameter are indicative of cell spreading and growth, while decreased colony circularity and increased aspect ratio are indicative of progression of MCF7 cells toward a more invasive morphology. These changes in MCF7 colony morphology can be partly attributed to the presence of BJ-5ta fibroblasts in the PF hydrogel matrix. BJ-5ta fibroblasts were observed to assume elongated morphologies within the hydrogel matrix, reminiscent of their mesenchymal phenotype. MCF7 cells localized in the vicinity of fibroblasts were observed to preferentially extend along the fibroblast orientation and form elongated colonies with increasing colony density, thereby leading to decreased colony circularity over time (Fig. 3B, see Supplementary Fig. S2). In the case of MDA-MB-231 cells (co-encapsulated with fibroblasts), increased cellular area (~600 μm^2^ on day 0 vs. ~1500 μm^2^ on day 28), increased cellular diameter (~30 μm on day 0 vs. ~45 μm on day 28), decreased cellular circularity (~0.3 on day 0 vs. ~0.2 on day 28) and increased cellular aspect ratios (~4.0 on day 0 vs. ~10.0 on day 28) over 28 days in culture were observed (Fig. 3L–O). In addition, cellular elongation length increased over time (~60 μm on day 0 to ~150 μm on day 28) (see Supplementary Fig. S3). These changes in MDA-MB-231 cells are indicative of cellular spreading and progression towards a more invasive morphology. Overall, the tumor-mimetic chips facilitated the long-term investigation of the morphological progression of two distinct breast cancer cell types in co-culture with fibroblasts and encapsulated within an ECM-mimic PEG-fibrinogen hydrogel matrix.

Cell growth and morphology within the tumor-mimetic chips is dependent in part on the degree of media perfusion from the vascular channels as well as oxygen permeability through the PDMS layers of the chips. The PDMS layers supporting the microvascular networks and tumor chamber are permeable to oxygen and hence allow diffusion of oxygen from the ambient atmosphere to the interior regions, in a thickness dependent manner^30,31^. Considering the uniform thickness of the top PDMS layer across the entire area of the central tumor chamber (and hence uniform oxygen permeability), variations in cell viability and growth can be primarily attributed to differences in media perfusion resulting from microvascular design variations and local shear rate differences. Specifically, in the above quantification, the distance of the MCF7 colonies from the surrounding vasculature was within a range of approximately 500–600 μm. Cells seeded beyond this region did not form distinct colonies and remained as single, rounded cells throughout the culture period, possibly in a quiescent state; the observed spatial differences are similar to tumor heterogeneity observed *in vivo*. Similar trends were also observed for MDA-MB-231 cells, with those within 500–600 μm of the vasculature having an elongated morphology and those beyond this distance appearing quiescent. No difference in viability of cell colonies was observed within 500–600 μm distance from the adjoining vasculature. Cell viability beyond this distance could not be quantified due to the inability of the Live/Dead dye to perfuse to the innermost regions of the tumor chamber and fluorescence signal being compromised by the presence of the central tubing and optical distortion near the port.

### Microfluidic pattern-dependent variation in vascular perfusion and morphological heterogeneity

Native tumors are often associated with abnormal vasculature in the surrounding microenvironment that is distinctly different in structure, integrity and morphology compared to healthy tissue vasculature. The tumor-associated vasculature is characterized by high tortuosity, leakiness, increased vessel permeability, presence of closed loops, sharp bends and even bidirectional, reversible flow in certain capillary channels^32,33^. These irregular microfluidic features and flow patterns combined with the high metabolic demand of the central tumor mass often creates zonal differences in perfusion of nutrients and cellular metabolites within various sections of the tumor mass. The relative degree of perfusion and mass transfer limitations within the central tumor mass often leads to the formation a central necrotic core surrounded by viable, proliferative cells in the peripheral zones adjoining the tumor vasculature^34^. Recapitulation of the cellular heterogeneity in 3D *in vitro* tumor models is essential in capturing the physiological complexities of native tumors^21^ and lends valuable information regarding tumor heterogeneity, cancer stem cell-like behavior, tumor hypoxia and efficacies of anti-cancer therapeutic strategies^35^.

**Figure 4:**
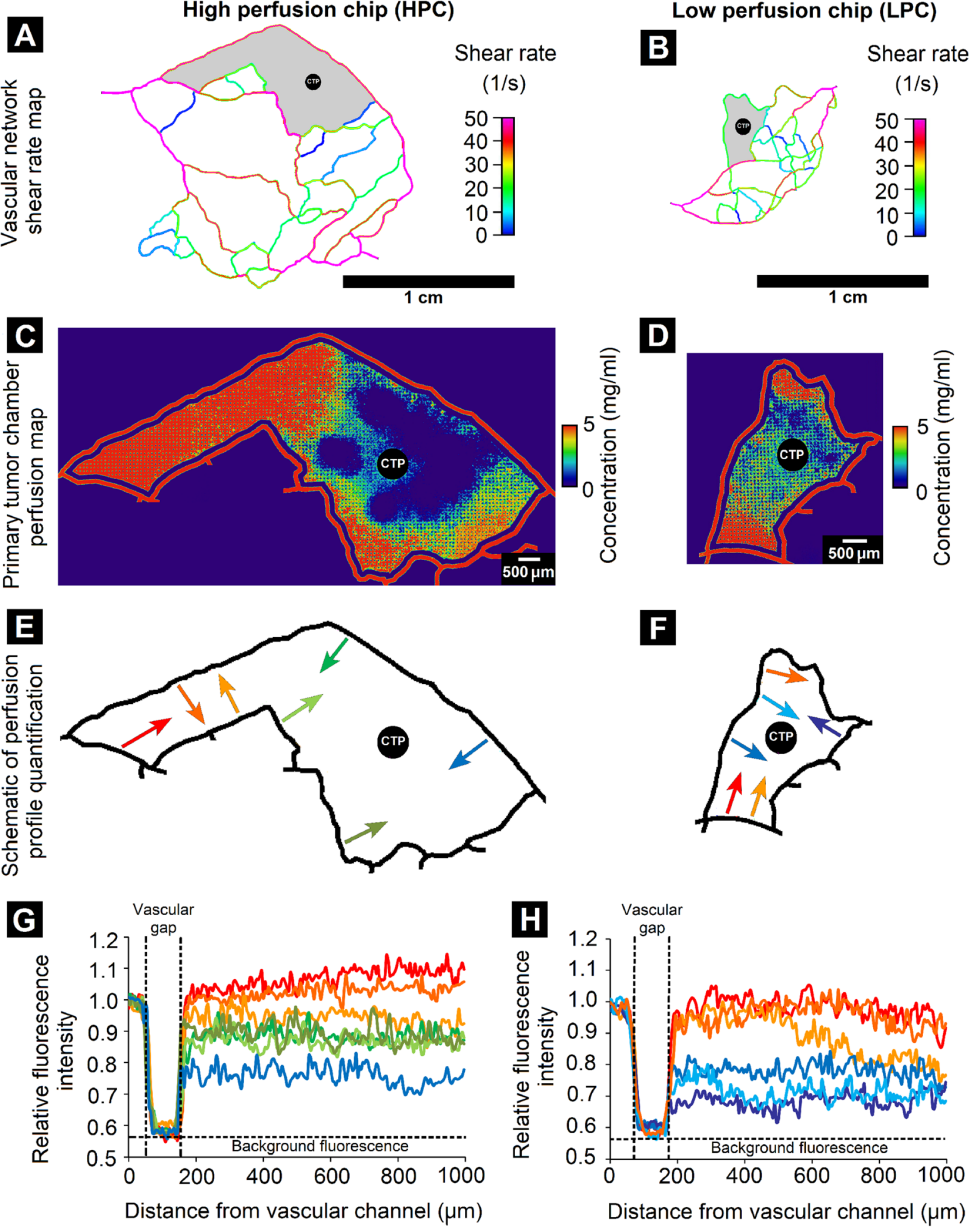
Variations in flow pattern and perfusion profiles within tumor-mimetic chips. Shear rate maps of (**A**) High perfusion chip (HPC) and (**B**) Low perfusion chip (LPC) obtained by computational fluid dynamics modeling reveal differences in local shear rates in various channels of the microfluidic network, specifically around the primary tumor chamber (grey region). Perfusion heat map of the primary tumor chamber of (**C**) HPC and (**D**) LPC revealed spatial differences in concentration of fluorescent TRITC-dextran perfused from the adjoining vascular channels into the tumor chamber (CTP indicates central tumor port). (**E,F**) Schematic representation of quantified perfusion profiles in HPC and LPC in different regions of the primary tumor chamber. Arrows indicate direction of vascular perfusion and profile measurement. Red and orange arrows represent relatively higher perfusion regions, green arrows represent intermediate perfusion regions and blue and purple arrows represent low perfusion regions. (**G,H**) Relative fluorescence intensity profiles in various regions of HPC and LPC corresponding to colored arrows in (**E**) and (**F**) Revealed prominent differences in perfusion capability in the various regions of the chips. Intensity values above 1.0 indicate relative accumulation or entrapment of fluorescent dye within the primary tumor chamber.

In this context, tumor-mimetic chips comprised of complex, intricate microvascular patterns that closely mimic native tumor vascular morphology were used to recapitulate zonal heterogeneity in perfusion and cancer cell morphology within the primary tumor chamber. Initially, the shear rates associated with the microvascular flow pattern within the tumor-mimetic chips were determined using computational fluid dynamic software and prominent differences between the two selected designs were observed. The high perfusion chip (HPC) provided higher shear rates (40–50 s^−1^) in various sections of the microfluidic network compared to that in the low perfusion chip (LPC) (10–20 s^−1^) (Fig. 4A,B). In order to assess the role of the microvascular architecture and shear variation on the perfusion phenomena within the tumor-mimetic chips, TRITC-dextran was used as a fluorescent diffusional marker to temporally visualize diffusion and quantify concentration gradients at different locations within the primary tumor chamber pre-seeded with cancer cells and fibroblasts in the PF hydrogel matrix. Zonal differences in perfusion were observed for both the HPC and LPC design. In the HPC design, a higher degree of TRITC-dextran perfusion was observed compared to that in the LPC design (Fig. 4C,D). The HPC design had a larger area of well-perfused regions (represented by red, orange and yellow) in the heat map as compared to the LPC design. This observation could be attributed in part to the higher shear flowrates in the adjoining vascular channels, and correspondingly higher volumetric flow rates, in the HPC design as compared to those in the LPC design, as well as the intrinsic mass transfer resistance presented by the encapsulated cells and supporting PF hydrogel matrix in the primary tumor chamber. Interestingly, in the HPC design, the well-perfused regions were located closer to the vascular channel inlet port of the chip. Analysis of the perfusion profile in the HPC design revealed that in multiple regions of the primary tumor chamber relatively high concentrations were maintained with increasing distance from vascular channel as compared to the LPC design (Fig. 4E–H). In certain regions of the HPC design, the relative fluorescent intensity was found to be higher in the tumor chamber than in the adjoining vascular channel, indicating the accumulation or entrapment of fluorescent TRITC-dextran within the PF hydrogel matrix and possible cellular uptake by encapsulated cancer cells and fibroblasts. In contrast, the LPC design featured multiple zones with sharp drops in tumor chamber concentration as compared to the adjacent vascular channel possibly due to effects of lower shear rates and correspondingly lower volumetric flow rates in the adjacent vascular channels.

Due to the zonal variations in perfusion and presence of concentration gradients of vascular media components, morphological differences in encapsulated cancer cells were also visualized within the primary tumor chamber of the tumor-mimetic chips (see Supplementary Fig. S4). In the high perfusion regions, MCF7 cells were able to form local colonies characteristic of their 3D phenotype (Supplementary Fig. S4B,C) while MDA-MB-231 cells formed took on elongated morphologies indicative of cell spreading (Supplementary Fig. S4D,E). However, in the low perfusion regions, both cell types appeared rounded, relatively dark and unhealthy, possibly indicative of a dormant, quiescent state due to lack of sufficient nutrient availability (Supplementary Fig. S4F–I). Fibroblasts present in the high perfusion regions had elongated morphologies but those present in the low perfusion regions appeared rounded and dormant. These cellular morphologies are consistent with that observed in native tumors where richly supplied regions contain viable, proliferative cells while poorly supplied regions experience necrotic cell death. Overall, the tumor-mimetic chips facilitated the recapitulation of the locational variations in vascular perfusion, concentration gradients and cellular characteristics that are characteristic of native tumors, thereby potentially providing the platform for future investigations in tumor heterogeneity.

### Assessment of anti-cancer drug efficacy and endothelial cytotoxicity

The ability of anti-cancer therapeutics to be transported from the site of delivery and reach the targeted tumor site is an important aspect of consideration in drug delivery systems. Prior to triggering their cytotoxic activity, delivered anti-cancer therapeutics need to overcome specific rate-controlling steps including the ability to perfuse through the complex, tortuous vasculature and reach the target tumor site, traverse the transendothelial barrier, interact extracellularly with the tumor parenchyma or be intracellularized by cancer cells^36^. Incorporation of these multiple transport steps in *in vitro* microfluidic cancer models is critical in assessing and optimizing the efficiency of delivery and therapeutic action of anti-cancer agents.

**Figure 5:**
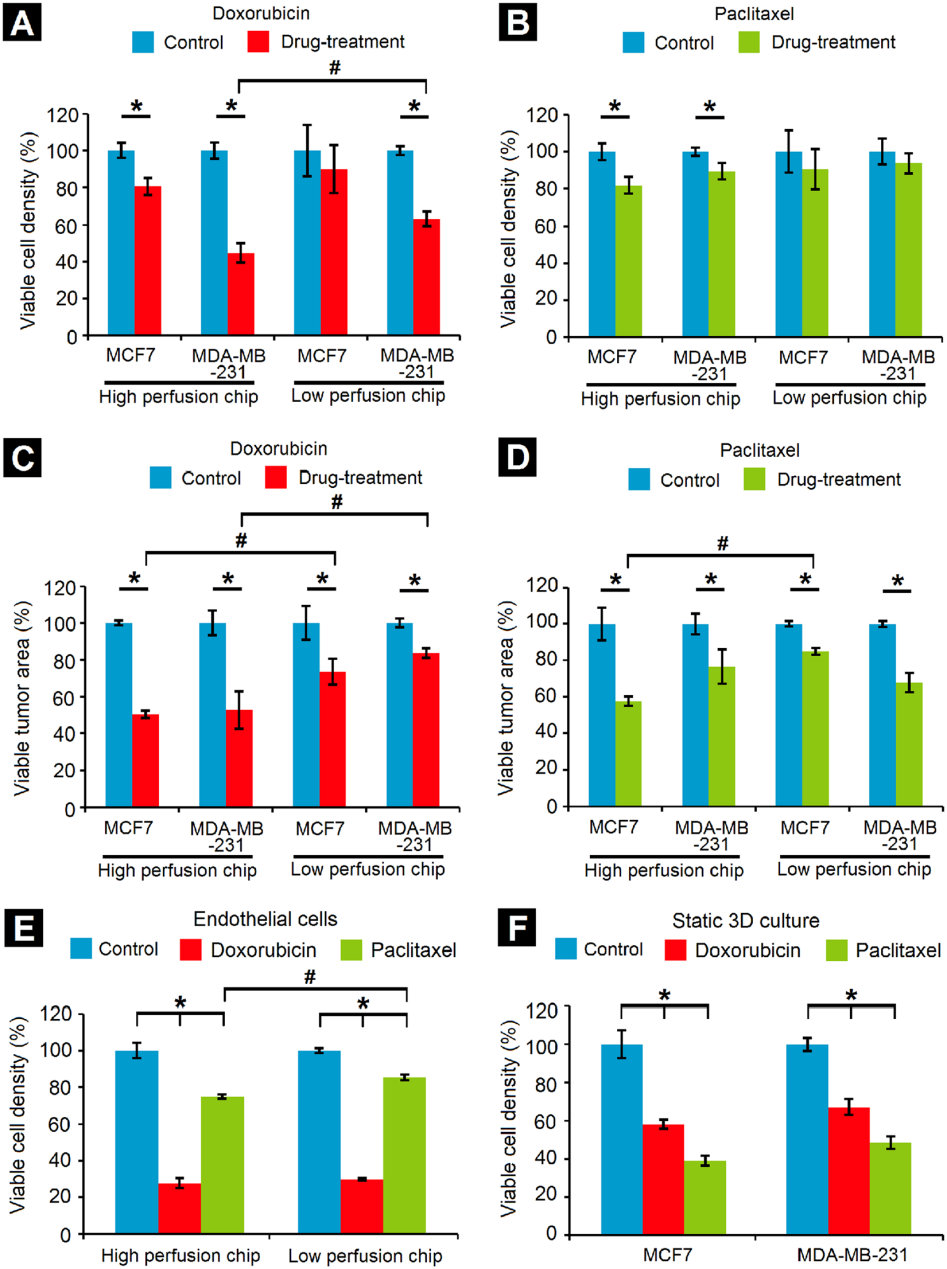
Drug-testing in tumor-mimetic chips. Reduction in viable cell density due to (**A**) Doxorubicin and (**B**) Paclitaxel. Viable MDA-MB-231 cell density (co-encapsulated with fibroblasts) following doxorubicin treatment was significantly lower in the HPC design as compared to the LPC design; this trend was not observed for the MCF7 cells (co-encapsulated with fibroblasts). No significant differences in viable cell density in either chip design were observed following paclitaxel treatment. Reduction in viable tumor area (area occupied by viable cells) due to (**C**) Doxorubicin and (**D**) Paclitaxel treatment revealed that doxorubicin caused a significant reduction in viable tumor area for both the cell lines in the HPC design as compared to the LPC design. Paclitaxel treatment caused a significant reduction in viable tumor area of MCF7 cells but not MDA-MB-231 cells. **(E)** Drug cytotoxicity on endothelial cells demonstrates greater cytotoxicity of doxorubicin as compared to paclitaxel in both chip designs. (**F**) Decrease in viable cell density of both cell lines in static 3D well plate culture (*Significant difference between control and drug treatment groups, p < 0.05; ^#^Significant difference between same cell type in different chip designs, p < 0.05, n = 3 independent chips per condition; for static 3D culture, n = 3 independent hydrogel constructs per condition, error bars represent standard deviation).

In order to investigate the effect of some of the rate-controlling factors on the delivery and efficacy of anti-cancer drugs, doxorubicin and paclitaxel were perfused through the vascularized tumor-mimetic chips containing the bioengineered breast tumor tissue. Initially, the tumor-mimetic chips were seeded with hBTECs and maintained under physiological flow (0.1 μL/min) for complete lumenization and the establishment of the tumor-mimetic vasculature. Next, MCF7 or MDA-MB-231 cells (cell density: 50 × 10^6^ cells/ml) were co-encapsulated with BJ-5ta fibroblasts (cell density: 10 × 10^6^ cells/ml) (cancer cell:fibroblast ratio = 5:1) within the ECM-mimic PF hydrogel matrix and cultured for 2 days prior to drug treatment. Doxorubicin or paclitaxel (10 μM) were perfused through the vascular inlet port and the resulting effect on cell viability in the tumor-mimetic chips was assessed 48 hours post-drug treatment. In general, doxorubicin appeared to have a greater cytotoxic effect on the cancer cells compared to paclitaxel in terms of viable cell density (Fig. 5A,B, see Supplementary Fig. S5A–F). In the HPC design, viable cell density was significantly reduced by both drug treatments for both cell lines, possibly due to higher penetration of drugs into the respective central tumor chambers of the tumor-mimetic chips. However, in the LPC design, only doxorubicin had an appreciable effect in reducing of MDA-MB-231 cell density. In addition, the density of MDA-MB-231 cells was significantly lowered in the HPC design compared to the LPC design following doxorubicin treatment. In terms of the viable tumor area (area occupied by the viable cancer cells, analogous to *in vivo* tumor shrinkage), significant reduction compared to the control was observed across all tested conditions (Fig. 5C,D). Specifically, viable tumor area of MCF7 cells (doxorubicin and paclitaxel) and MDA-MB-231 cells (doxorubicin only) was significantly reduced in the HPC design compared to the LPC design, possibly due to potentially higher penetration of drug in the primary tumor chamber and the mechanism of drug action (see Supplementary Fig. S6).

Further assessment of the associated cytotoxic effects on hBTECs revealed significantly lower viability and density of endothelial cells following doxorubicin treatment as compared to paclitaxel treatment in both the HPC and LPC chip designs (Fig. 5E, see Supplementary Fig. S5G–I). The toxicity of doxorubicin is well-known and it causes endothelial cell death via apoptosis^37,38^. Specifically, whereas doxorubicin’s anti-cancer activity is associated with DNA binding and through acting as a topoisomerase-2β antagonist, damage to endothelial cells and cardiomyocytes is believed to be related to the formation of reactive oxygen species and oxidation of cellular membranes, including that of the mitochondria. Hence, the tumor-mimetic platform facilitates not only the perfusion-dependent quantification of drug action on cancer cells, but also the assessment of side effects on associated endothelial cells. Taken together, these results provide a relative perspective on the interdependent role of multiple tumor microenvironmental factors in determining the efficacy of anti-cancer drugs.

Parallel drug studies were conducted in well plates with static 3D cultures of cancer cells co-encapsulated with BJ-5ta fibroblasts within PF hydrogels, but in the absence of endothelial cells or dynamic flow conditions, which are not available in static assays and are one of the primary motivations for the present study. Interestingly, both doxorubicin and paclitaxel had a much greater cytotoxic effect on MCF7 and MDA-MB-231 cells in these static 3D culture conditions compared to that under the flow conditions in the tumor-mimetic chips (Fig. 5F, see Supplementary Fig. S7). These differences in drug response highlight the importance of incorporating a fluidic, tumor-associated vascular network surrounding the central tumor tissue on the delivery and efficacy of the anti-cancer drugs.

## Discussion

In recent studies, various tumor-on-a-chip systems have been developed with multiple configurations in order to investigate specific cancer-associated phenomena including tumor-endothelial interactions^39^, intravasation and extravasation^40,41^, tumor angiogenesis^42^ and drug testing applications^14,43–45^, amongst others. However, the majority of microchip systems are simplistic in design and reductionist in approach^15^, thereby limiting their physiological context with respect to recapitulation of the complex architecture and tumor microenvironment (TME). In this study, we have established a microfluidic platform where multiple levels of physiological complexity are incorporated by maintaining an intricate network of the tumor-mimetic vasculature in co-culture with cancer cells and stromal fibroblasts within an ECM-mimic matrix. As a major advancement over other microchip systems, cancer cells can be maintained in long-term culture (at least 28 days) within the tumor-mimetic chips, enabling the investigation of morphogenic tumor progression over time and future testing of metronomic drug dosing^46^.

Cancer cell intravasation into surrounding tumor vasculature and extravasation into secondary sites are key events in the complex metastatic cascade^1,47^. The ability to monitor cancer cell invasiveness and migratory behavior, in the presence of endothelial interactions, is a valuable and desired feature of 3D *in vitro* models. In this context, the ability of encapsulated breast cancer cells to intravasate from the primary tumor chamber into the adjoining lumenized microvascular channels and invade into the secondary tumor chambers was observed and quantified in long-term 3D culture within the tumor-mimetic chips. Metastatic MDA-MB-231 cells were observed to undergo transendothelial migration (TEM) from the primary to the secondary tumor chambers in long-term 3D culture (see Supplementary Fig. S8A–C). Cellular intravasation increased from day 0 to 7, but decreased at later time points (see Supplementary Fig. S8D), possibly due to low shear rate in vascular channel between the primary and secondary tumor chambers, reduced media diffusion and subsequent cell death in the primary tumor chamber. Additionally, extravasated cell density in the secondary tumor chamber and cellular invasion distance were also observed to increase over time (see Supplementary Fig. S8E,F). In contrast, non-metastatic MCF7 cells remained localized within the primary tumor chamber without displaying any migratory tendency. These observations are reflective of the invasive and metastatic behavior of the MDA-MB-231 cancer cells as is observed in *in vivo* systems. The ability to visualize and quantify the 3D migratory behavior of cancer cells under dynamic flow conditions and ECM-mimetic microenvironment highlight the usefulness of the chip-based tumor-mimetic platform in assessing the invasiveness and metastatic potential of other cancer cells in the future.

The dynamic flow conditions and fluidic exchange between the vascular channels and tumor chambers facilitate investigation of tumor-endothelial interactions, tumor cell migration and the development of locational heterogeneity in vascular perfusion, concentration gradients and cellular presentation. Additionally, the recapitulation of key physiological complexities of the TME provides contextual information of drug action and associated cytotoxicity on cells within the tumor-mimetic chips. An innovative aspect of the tumor-mimetic chips is the use of PEG-fibrinogen (PF) hydrogels as the ECM-mimic matrix encapsulating cancer cells and fibroblasts. Fibrinogen is known to be exuded exogenously from blood plasma into the surrounding ECM and endogenously secreted and deposited in the ECM by cancer cells^48–50^. Stromal fibrinogen induces tumorigenic growth, transendothelial migration and metastatic progression of cancer cells and also promotes angiogenic growth and signaling mechanisms of endothelial cells^51–53^. Employing PF hydrogel as the biomimetic matrix for 3D cell culture provides the ability to modulate the biochemical and mechanical characteristics of the matrix and tune them to that of the native TME^54,55^.

The stiffness of the PF hydrogel matrix can be modulated by the addition of excess PEGDA (additional 1% or 2% w/v) within the polymer precursor prior to crosslinking, which leads to increased crosslinking density and increased Young’s moduli of the bulk matrix. MCF7 or MDA-MB-231 cells and BJ-5ta fibroblasts co-encapsulated within PF matrices of increasing PEGDA concentration (PF + 1%P and PF + 2%P hydrogels) exhibited increasing Young’s moduli (see Supplementary Fig. S9). The matrix composition and stiffness also induced changes in the morphological progression of encapsulated cells over time (see Supplementary Fig. S10). Immunofluorescence staining and imaging of co-cultured cancer-fibroblast PF hydrogel constructs revealed a high degree of proliferation, cell spreading and expression of characteristic cellular markers (see Supplementary Fig. S11), thereby demonstrating the ability of PF hydrogel matrix in supporting the growth and morphological expression of cells in 3D culture. Co-culture of stromal fibroblasts and supporting cell types with cancer cells in 3D microenvironments allow for investigation of vital intercellular interactions and bidirectional signaling mechanisms involved in tumor progression and malignancy^4,6^. Overall, the use of PF hydrogel matrix for encapsulation and 3D culture of cancer cells and fibroblasts presents an interesting opportunity in recapitulating the native tumor ECM and also for investigation of long-term 3D cellular behavior.

The ability of the tumor-mimetic chips in simulating key features of the TME also presents an interesting opportunity in testing the efficacy and safety of anti-cancer therapeutics with a higher degree of physiological relevance than that provided by other microchip systems. Through previous studies, it is well known that cancer cells in 3D culture have a higher degree of chemoresistance than those in standard 2D culture^6,56^. However, even within 3D culture models, the incorporation of various degrees of physiological complexities including the presence of stromal fibroblasts and dynamic vascular flow conditions can also elicit prominent differences in anti-cancer drug efficacy. These differences in drug action can be attributed in part to a number of factors: 1) the design-dependent variations of shear flow and concentration gradients within the tumor-mimetic chips that dictate the drug exposure ultimately experienced by the cancer cells in different locations of the primary tumor chamber, 2) the state of proliferation and metabolic activity of the two cell types, 3) the differences in mechanism of drug action and 4) the rate of drug transport across the trans-endothelial barrier and rate of intracellular drug uptake. Specifically, doxorubicin is known to intercalate with DNA, disrupt topoisomerase-II-mediated DNA repair and generate reactive oxygen species leading to oxidative stress and apoptotic cell death^57^. Since doxorubicin can bind to DNA at any point in the cell cycle, its cytotoxic effects are persistent and can be observed in subsequent cell cycling stages after removal of excess doxorubicin. In contrast, paclitaxel binds to β-tubulin subunits of microtubules, stabilizing them and preventing them from disassembly during metaphase spindle formation, thereby causing mitotic arrest in the G2/M phase of the cell cycle and subsequent cell death^58^. Since paclitaxel action is cell-cycle dependent, only small fraction of cells may be susceptible to its action during the time frame of infusion. Additionally, paclitaxel has a much greater volume of distribution compared to doxorubicin due to its physiochemical properties and is rapidly and more widely distributed through the tumor parenchyma. In the tumor-mimetic chips, paclitaxel had a much lower cytotoxic effect on the cancer cells compared to that of doxorubicin. This could be attributed in part to the comparative differences in the ability of paclitaxel and doxorubicin to extravasate through the endothelial layer and diffuse through the PF hydrogel matrix encapsulating the cancer cells. The proliferation state of encapsulated cancer cells and their susceptibility towards drug action mechanism could be influential factors in their chemosensitivity. This would also explain the differences in post-treatment cytotoxicity of the drugs observed on endothelial cells. The hBTECs were fully lumenized and stabilized in the microvasculature of the chips and hence not in a state of active cell division. Hence, these cells were affected by paclitaxel to a much lesser degree than doxorubicin.

Despite the demonstrated capabilities, the tumor-mimetic chips suffer from certain limitations. In addition to the inherent differences between cancer organ-on-a-chip and animal models^59–61^, there are also some specific considerations with regard to the use of PF hydrogel matrix within the chips. The PF hydrogels tend to swell within buffer or media after light-mediated crosslinking and attain an equilibrium state. However, in this chip system, the hydrogel matrix encapsulating cells is spatially confined in all directions by the PDMS side and upper walls and the glass bottom within the primary tumor chamber, thereby exerting reverse pressure on the hydrogel matrix. This phenomenon potentially causes the encapsulated cells to experience more pressure than they would within a free-floating unconfined hydrogel mass. The pressure exerted by the hydrogel mass within the primary tumor chamber could also potentially provide additional mass transfer resistance to the media being perfused through the vascular channels, thereby limiting long-term viability of cells seeded further away from the vascular channels. In addition, the perfusion of staining reagents and washing solutions deep into the primary tumor chamber also proved challenging due to the pressure buildup of the PF matrix. In spite of these limitations, the tumor-mimetic chips allow for examining many of the complex pathophysiological attributes of TME, thereby reducing animal use. Although this system cannot replace complexity of the *in vivo* condition, the chips can be readily implemented by other labs via appropriate commercial or collaborative arrangements to test various hypothesis prior to conducting *in vivo* studies. They also provide improved control and reproducibility over experimental conditions and could potentially reduce the burden of subsequent *in vivo* studies.

Overall, these cancer-on-chip platforms facilitate the investigation of anti-cancer drug efficacy and cytotoxicity with a greater degree of physiological context (in the presence of dynamic and pattern-dependent flow) than that available in static 3D systems. The presence of an intricate network of complex and abnormal microvasculature, that is found surrounding tumor tissues, provides shear flow variances and perfusion differences in the flow of metabolites and drugs. The presence of the PF matrix simulates native tumor ECM matrices that provide mass-transfer limitations to the diffusion of different molecules. The presence of fibroblasts as a supportive secondary cell type provides a greater degree of physiological context with regards to *in vivo* tumors. Specifically, the microfluidic platform can provide important information regarding the distribution of drug compounds through the vascular network and within the central tumor chamber and subsequent effects of drug action on encapsulated cancer cells. Comprehensively, the complex interplay and influence of all these parameters significantly enhance the ability of this platform to provide predictive information of drug action with a higher degree of accuracy compared to traditional 2D and static 3D model systems.

## Conclusions

This study presents a microfluidics-based tumor-mimetic chip system for the long-term 3D co-culture of cancer cells and fibroblasts within an ECM-mimic hydrogel matrix along with an intricate, tumor-associated lumenized vasculature for the recapitulation of the native TME *in vitro*. This represents an advance over existing platforms and provides a novel approach to test the effects of different therapeutic agents and determine their exposure-response relationships under dynamic flow conditions. Variations in vascular architecture and design enabled the replication of key features of the native TME including locational heterogeneity, concentration gradients and cellular morphology. Finally, the tumor-mimetic chips facilitated the observations of cell migration, assessment of efficacy and toxicity of anti-cancer therapeutics with a higher degree of physiological relevance relative to conventional systems.

## Methods

### Fabrication of tumor-mimetic chips

The HPC and LPC designs for the tumor-mimetic chips were obtained from digitized images of mouse vasculatures similar to our previous studies^26,62,63^. The vessel wall adjacent to the tumor growth region was modified in AutoCAD to engineer the LPC and HPC devices. The designed networks were used to fabricate high resolution photomasks (Advanced Reproduction Corporation). The photomasks were used to pattern the photoresist (SU-8) on silicon (Si) wafers to generate masters for the microfluidic device. The process includes, a) spin coating SU-8 to achieve desired height of the pores, b) exposure and developing of SU-8, c) spin coating of 2^nd^ layer of SU-8 to achieve 100 µm total height, d) alignment of 1^st^ layer of SU-8 with second mask containing fluidic channels, f) pattern exposure and developing of SU-8. Sylgard 184 PDMS (Dow Corning) was poured over the developed master to generate replica cast devices in PDMS. Through holes, defining the inlets and outlets were punched using a 1.5 mm biopsy punch. For injection of tumor cells, a 30 gauge needle was used to punch holes in the tumor area with a stereo microscope for alignment. The PDMS devices and a pre-cleaned glass slide were cleaned using oxygen plasma treatment (150 mTorr, 50 W, 20 s) prior to bonding. Tygon Microbore tubing (O.D. 0.06″ and I.D. of 0.02″) served as the connecting ports for fluidic interfacing to a programmable syringe pump.

### Cell culture and maintenance

MCF7 (ATCC^®^ HTB-22^™^) and MDA-MB-231 (ATCC^®^ HTB-26^™^) human breast cancer cells and BJ-5ta (ATCC^®^ CRL4001^™^) normal human foreskin immortalized fibroblasts were obtained from ATCC (Manassas, VA). Cancer cells were maintained in Dulbecco’s Modified Eagle’s Medium (DMEM) (Gibco) supplemented with 10% fetal bovine serum (FBS) (Atlanta Biologicals), 1% (v/v) non-essential amino acids (Lonza), 1% (v/v) penicillin/streptomycin, 1% (v/v) Glutamax (Gibco) and 1% (v/v) sodium pyruvate. BJ-5ta cells were maintained in 4 parts of DMEM containing 4 mM L-glutamine, 4.5 g/L glucose and 1.5 g/L sodium bicarbonate, 1 part of Medium 199 supplemented with 0.01 mg/ml hygromycin B and 10% FBS. Human breast tumor-associated endothelial cells (hBTECs) were obtained from Cell Biologics (Chicago, IL) and were maintained in Human Endothelial Cell Medium (Cell Biologics) supplemented with 0.5 ml VEGF, 0.5 ml heparin, 0.5 ml EGF, 0.5 ml hydrocortisone, 5.0 ml L-glutamine, 5.0 ml antibiotic-antimycotic solution, 10.0 ml endothelial cell supplement and 50.0 ml FBS.

### Formation and characterization of tumor-mimetic vasculature

Fabricated chips were initially degassed and coated with human fibronectin solution (Sigma-Aldrich) (100 μg/ml) for 3 hours and with a gelatin-based coating solution (Cell Biologics) for 30 minutes prior to cell seeding. hBTECs were resuspended in endothelial media at 50 × 10^6^ cells/ml, manually flowed in through the inlet port and allowed to attach overnight. The cells were maintained under continuous flow (0.1 μL/min) via syringe pump (KD Scientific) for at least 2 days to form lumenized vasculature. For immunostaining of hBTECs, cells were washed with PBS to remove media, fixed with 4% paraformaldehyde solution for 10 minutes, permeabilized with PBS-T for 10 minutes and blocked with blocking buffer for 3 hours at room temperature. Cells were stained with CD31-FITC (Invitrogen, 1:50 dilution) or αSMA (mouse primary antibody, Abcam, 1:100 dilution) overnight. Next, cells were stained with Alexa Fluor 568 Phalloidin (Invitrogen, 1:200 dilution) or Alexa Fluor 568 goat anti-mouse secondary antibody (Invitrogen, 1:200 dilution) and Hoechst 33342 (1:200 dilution) for 2 hours. After washing with PBS, fluorescence images were obtained using confocal microscopy (Nikon AI Confocal Scanning Laser Microscope) to obtain z-stacks. Fluorescence images were analyzed using ImageJ software (NIH, Version 1.51) to obtain morphological characteristics of endothelial cells similar to previous studies (see Supplementary Methods)^21,54^.

### Maintenance of cancer-fibroblast co-culture

PEG-fibrinogen (PF) was synthesized and characterized as described previously^64^. Tumor-mimetic chips lumenized with hBTECs were used for co-culture of cancer cells with fibroblasts within PF hydrogels. MCF7 or MDA-MB-231 cells (cell density: 50 × 10^6^ cells/ml) were resuspended with BJ-5ta fibroblasts (cell density: 10 × 10^6^ cells/ml) within the PF hydrogel precursor. The PF precursor containing cancer cells and fibroblasts was perfused through the central tumor port into the primary tumor chamber and crosslinked via exposure to visible light for 2 minutes. Fresh media was perfused at 0.1 μL/min every 6 hours within the chips. The chips were imaged every 7 days under phase contrast microscopy and images were analyzed via ImageJ software (NIH, Version 1.51) to evaluate morphological features of cancer cells as shown previously^51^. In order to assess viability of cells within tumor-mimetic chips, Live/Dead^®^ cell viability stain (Invitrogen, Carlsbad, CA) (calcein AM: 0.5 μL/mL and ethidium homodimer-1: 2 μL/mL in PBS) was perfused within the chips, incubated for 20 minutes, washed with additional PBS and imaged under fluorescence (FITC filter cube: Excitation: 480 ± 10 nm, Emission: 520 ± 10 nm, TRITC filter cube: Excitation: 555 ± 10 nm, Emission: 600 ± 20 nm) via a Nikon Ti inverted microscope. The number of live and dead cells were counted manually using ImageJ software^54,55^.

In order to modulate PF hydrogel matrix characteristics, PEGDA (250 mg/ml solution in PBS) was added to the PF hydrogel precursor at 1% or 2% w/v prior to cell encapsulation. Immunofluorescence staining and confocal imaging of cancer-fibroblast co-culture within PF hydrogels were conducted as described above. Ki67 (rabbit primary, Abcam, 1:100 dilution), E-cadherin (rabbit primary, Cell Signaling, 1:50 dilution) and vimentin (mouse primary, Cell Signaling, 1:100 dilution) were used as primary antibodies and Alexa Fluor 488 goat anti-rabbit (Invitrogen, 1:200 dilution), Alexa Fluor 568 Phalloidin (Invitrogen, 1:200 dilution), Alexa Fluor 568 goat anti-mouse (Invitrogen, 1:200 dilution) were used as secondary antibodies.

### Mechanical characterization of cancer-fibroblast co-cultures

The Young’s moduli of PF hydrogel matrices of varying composition were assessed by subjecting samples to parallel-plate compression testing using a CellScale Microsquisher^®^ system and analyzing via associated SquisherJoy software as shown earlier^51^. Briefly, acellular or cell-laden hydrogel constructs (diameter = 4 mm, thickness = 600 μm) were maintained in 3D culture for 7 days prior to mechanical testing. These constructs were then loaded onto the Microsquisher^®^ platform, maintained at 37 °C in PBS, preconditioned for compression testing and made to undergo 3 cycles of compression and relaxation at a rate of 5 μm/s for a minimum of 15% strain. The force-displacement data obtained from the compression test were converted to stress-strain curves and the lower portion of the curve (5–15% strain) was used to obtain a linear regression line and estimate the Young’s moduli of the hydrogel constructs.

### Characterization of diffusion gradients

Tumor-mimetic chips were seeded with hBTECs and maintained under dynamic flow to form lumenized vasculature. MCF7 cells and BJ-5ta fibroblasts were co-encapsulated in the PF hydrogel matrix within the primary tumor chamber and maintained in culture overnight. Fluorescent TRITC-dextran (Sigma-Aldrich, molecular weight: 4400 Da, 1 mg/ml in dH_2_O) was perfused through chips at 1 μL/min for 2 hours. The chips were imaged under fluorescence (TRITC filter cube: Excitation: 555 ± 10 nm, Emission: 600 ± 20 nm) microscope and the images were analyzed using ImageJ software to generate heat maps of the concentration profile within primary tumor chamber. A blank chip perfused with distilled water was used as a control for background subtraction. Fluorescent intensity values within the tumor chamber were normalized to that of TRITC-dextran in the inlet vascular channel. In addition, the images were also analyzed to estimate the diffusion gradients in specific regions of the HPC and LPC chip designs using MS Excel. Phase contrast images of various regions of the cell-seeded microfluidic devices were also obtained to visualize morphological heterogeneity.

### Anti-cancer drug testing

Tumor-mimetic chips lumenized with hBTECs and seeded with cancer cells and fibroblasts in the PF hydrogel matrix were maintained in culture for 2 days prior to drug treatment. Doxorubicin hydrochloride and paclitaxel (Euroasian Chemicals) were dissolved in dimethylsulfoxide (DMSO) at 10 mM concentration. The stock drug solutions were diluted to 10 μM in hBTEC media prior to infusion in the chips. The drugs were perfused via syringe pump at 1 μL/min for 19 minutes (for HPC design chip) or 7.5 minutes (for LPC design chip) and incubated for 4 hours. Fresh media was perfused in the chips to remove excess drug and the chips were maintained in culture for a further 48 hours prior to Live/Dead cell viability staining. Fluorescence images of stained regions of the chips were taken and a relative number of live cells (viable cell density) and area occupied by the live cells (viable tumor area) were analyzed by ImageJ software. Chips with media perfusion were kept as controls and chips with 0.1% DMSO were kept as vehicular controls. For static 3D culture drug testing, cancer cells and fibroblasts were co-encapsulated at 5:1 ratio within PF hydrogel discs of diameter 4 mm and thickness 600 μm. Drug solutions were added to hydrogel discs incubated in well plates for 4 hours prior to media change. Live/dead viability staining was conducted 48 hours post-drug treatment and fluorescence images obtained from hydrogel discs were quantified manually in ImageJ software.

### Statistical analysis

All statistical analysis was performed using Minitab 17 Statistical Software (Minitab Inc.). After checking for normality of distribution, One-way ANOVA with Tukey’s family error rate of 5% was used to evaluate statistical significance between multiple groups, with the assumption of equal variance and equal sample size between groups. In case of unequal variance between groups, the Games-Howell post-hoc test was employed following the ANOVA analysis. Unless otherwise indicated, p < 0.05 was considered statistically significant.

### Data availability

The datasets generated during and/or analyzed during the current study are available from the corresponding author on reasonable request.

## Electronic supplementary material

Supplemental Figures and Methods

Supplementary Movie S1
