# Supplementary material for: A Microvascularized Tumor-mimetic Platform for Assessing Anti-cancer Drug Efficacy
Source: Sci Rep. 2018 Feb 16;8:3171. doi: 10.1038/s41598-018-21075-9 (PMC5816595; doi:10.1038/s41598-018-21075-9)
Supplement: Supplementary file 1 — Supplemental Figures and Methods [file 41598_2018_21075_MOESM1_ESM.pdf]

SUPPLEMENTARY FIGURES AND METHODS

A Microvascularized Tumor-mimetic Platform for Assessing Anti-cancer Drug Efficacy

Shantanu Pradhan[1], Ashley M. Smith[2], Charles J. Garson[2], Iman Hassani[1], Wen J. Seeto[1], Kapil Pant[2], Robert D. Arnold[3], Balabhaskar Prabhakarpandian[2], Elizabeth A. Lipke[1*]

[1]Department of Chemical Engineering, Auburn University, Auburn AL 36849
[2]Biomedical Technology, CFD Research Corporation, Huntsville, AL 35806
[3]Department of Drug Discovery and Development, Auburn University, Auburn, AL, 36849

Supplementary Figures:

Supplementary Movie S1. Lumenized microvasculature. 3D rotated view of microvascular bifurcation junction with hBTECs (CD31, green; F-actin, red; nuclei, blue) forming lumenized structure with complete channel wall coverage (Scale bar = 100µm).

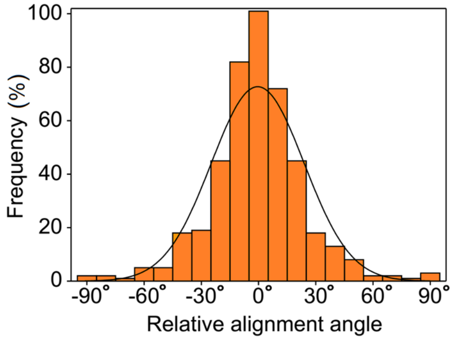

Supplementary Figure S1. hBTEC orientation in microvascular channels. Quantification of relative alignment angle of hBTECs revealed a high percentage of cells aligned to within ±30° of the flow direction in various sections of the microvascular network (n = 444 cells from 3 independent chips).

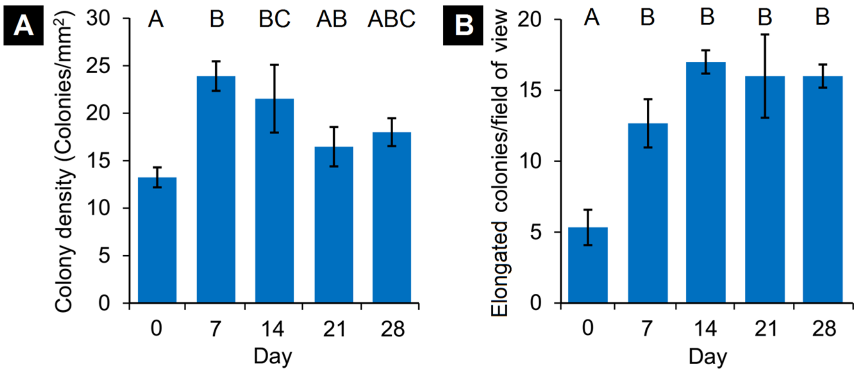

Supplementary Figure S2. MCF7 colony morphology over time. (A) Individual MCF7 cells form local colonies within tumor-mimetic chips leading to an initial increase in colony density; however, as individual small colonies merge together to form larger colonies, the colony density is marginally reduced at later time points. **(B)** MCF7 colonies are influenced by BJ-5ta fibroblasts to form elongated morphologies that gradually increase in number over time. Groups having different letters have significantly different means ($p < 0.05$); n = minimum 15 MCF7 colonies from 3 independent chips per time point, error bars represent standard deviation.

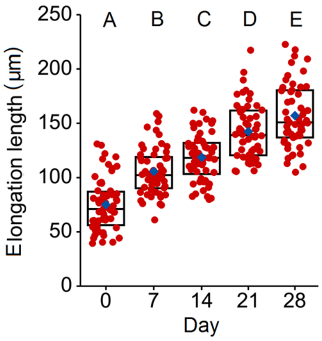

Supplementary Figure S3. MDA-MB-231 cell elongation over time. MDA-MB-231 cells displayed an elongated morphology in 3D culture within tumor-mimetic chips with gradually increasing elongation length over 28 days in culture. Red points denote individual MDA-MB-231 cells and blue diamonds represent mean of respective groups. Rectangular boxes represent upper quartile, median and lower quartile of respective group. Groups having different letters have significantly different means ($p < 0.05$); n = minimum 50 MDA-MB-231 cells from 3 independent chips per time point.

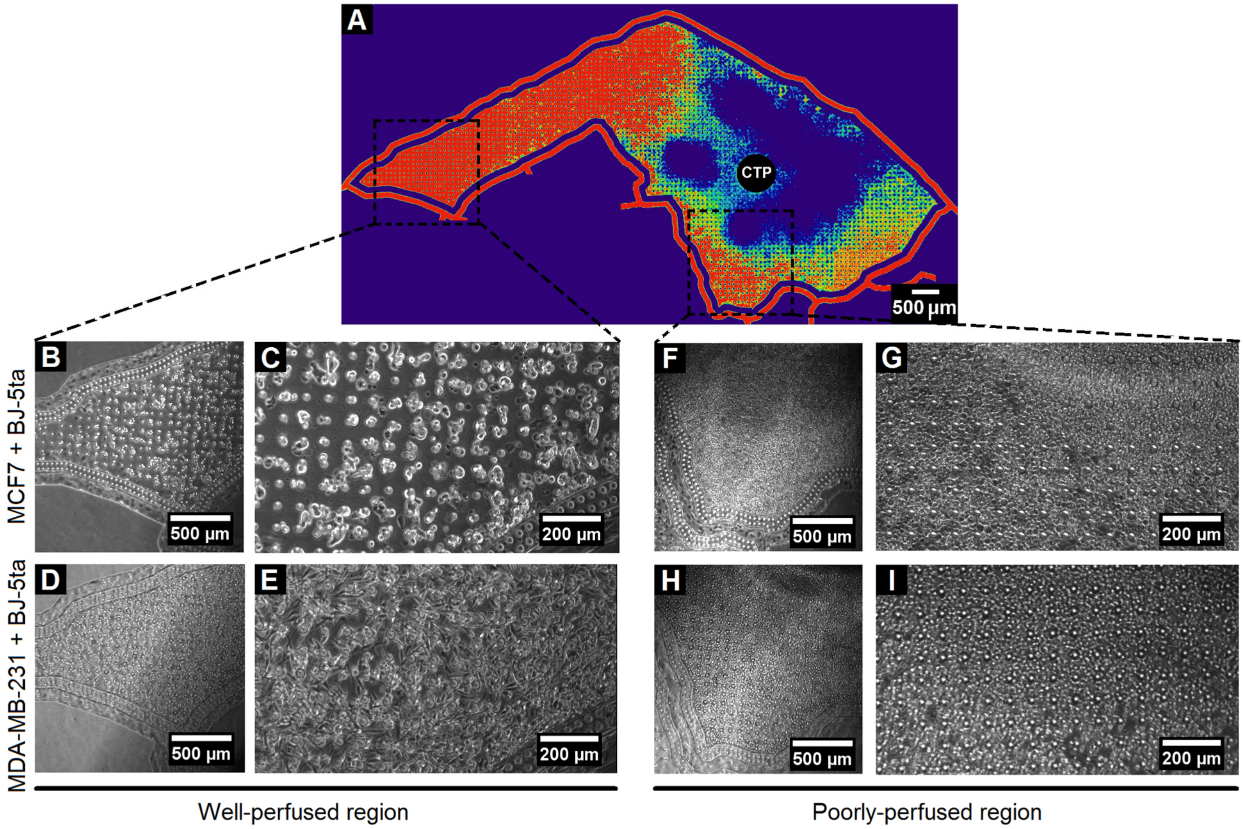

Supplementary Figure S4. Morphological heterogeneity of cancer cells. (A) Spatial variation in the relative ability of media components to be perfused into the primary tumor chamber of the high perfusion chip led to differences in morphology of breast cancer cells and fibroblasts co-cultured over time (CTP indicates central tumor port). **(B-C)** MCF7 cells (co-encapsulated with fibroblasts) formed local colonies and **(D-E)** MDA-MB-231 cells (co-encapsulated with fibroblasts) displayed an elongated morphology within the well-perfused regions of the primary tumor chamber owing to sufficient nutrient availability. **(F-G)** MCF7 cells and **(H-I)** MDA-MB-231 cells exhibited a rounded morphology and potentially quiescent 3D behavior in the poorly-perfused regions of the primary tumor chamber.

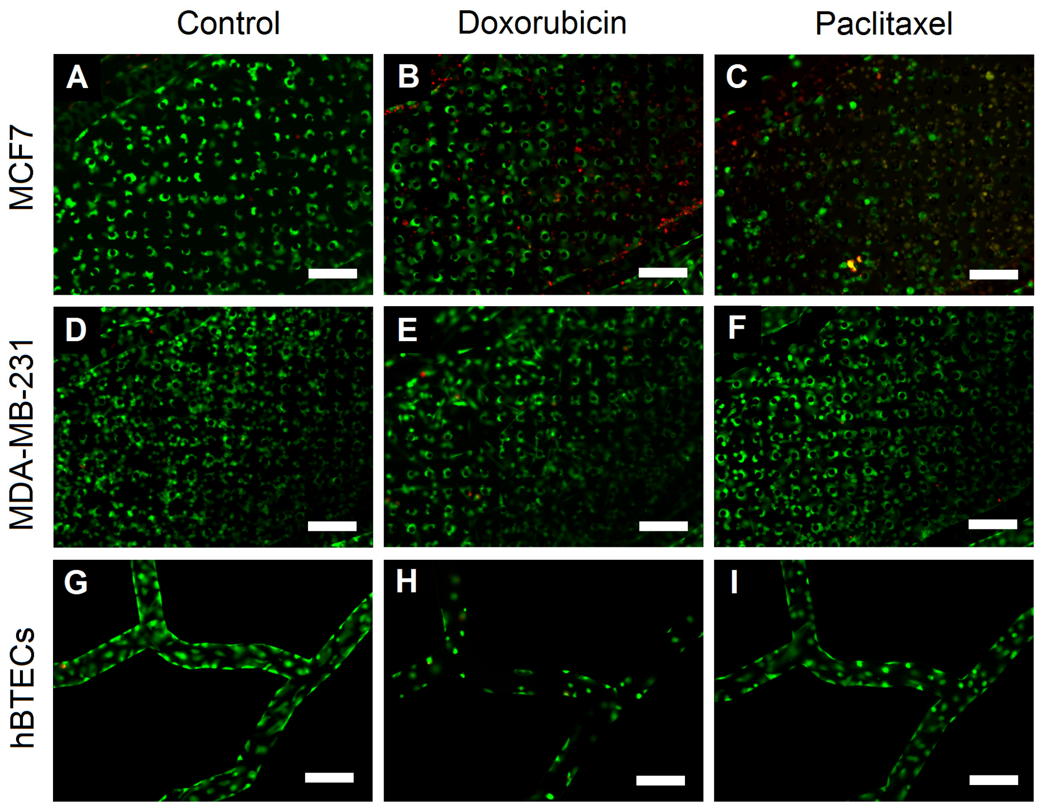

Supplementary Figure S5. Effect of drug treatment on cell viability within tumor-mimetic chips. Live/dead staining of **(A-C)** MCF7+fibroblast cells, **(D-F)** MDA-MB-231+fibroblast cells and **(G-I)** human breast tumor-associated endothelial cells 48 hours post-drug treatment in the high-perfusion design (HPC) chip (calcein AM, green; ethidium homodimer, red). Scale bar = 200 μm.

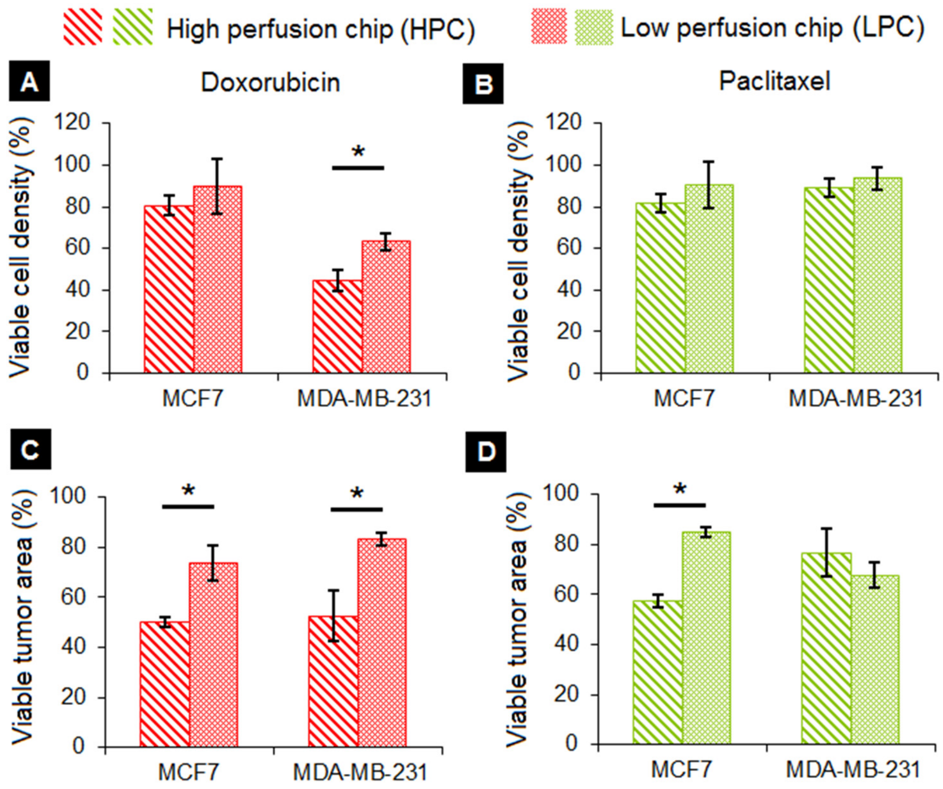

Supplementary Figure S6. Comparison of high perfusion and low perfusion chip design with respect to drug action. In terms of cell viability, **(A)** doxorubicin treatment significantly reduced MDA-MB-231+fibroblast viable cell density in the HPC design compared to the LPC design; this trend was not visible for MCF7+fibroblast cells. **(B)** Paclitaxel treatment did not elicit any significant differences in viable cell density in either chip design. **(C)** Viable tumor area was significantly lower in the HPC design as compared to LPC design for both cancer cell types under doxorubicin treatment. (D) Viable tumor area for MCF7 cells only, and not MDA-MB-231 cells, was significantly lower in the HPC design as compared to LPC design under paclitaxel treatment. (* Significant difference between HPC and LPC design groups, $p<0.05$; n = 3 independent chips per condition, error bars represent standard deviation).

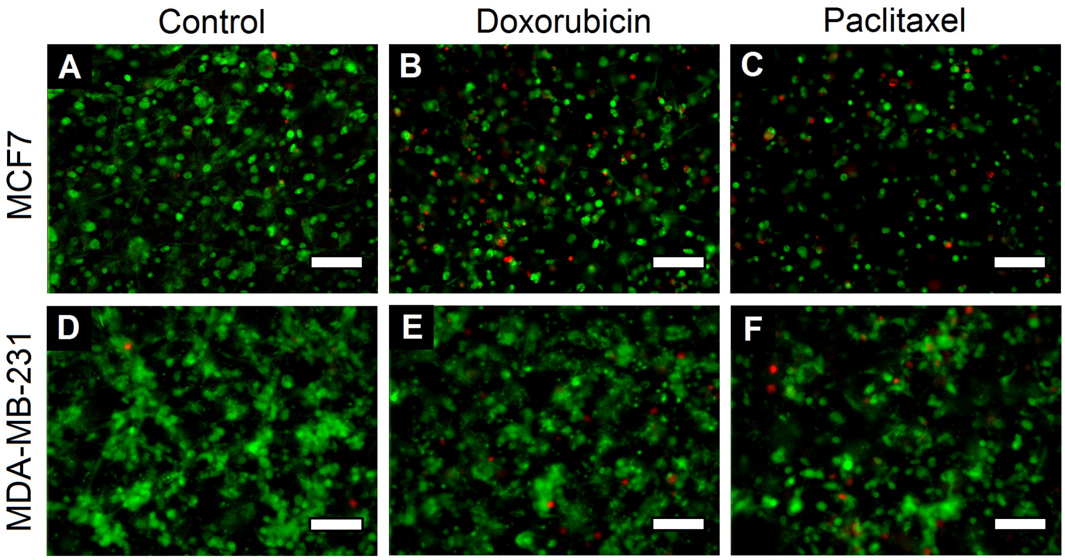

Supplementary Figure S7. Effect of drug treatment on cell viability within static 3D hydrogel cultures. Live/dead staining of **(A-C)** MCF7+fibroblast cells, **(D-F)** MDA-MB-231+fibroblast cells 48 hours post-drug treatment within static 3D PF hydrogels (calcein AM, green; ethidium homodimer, red). Scale bar = 200 μm.

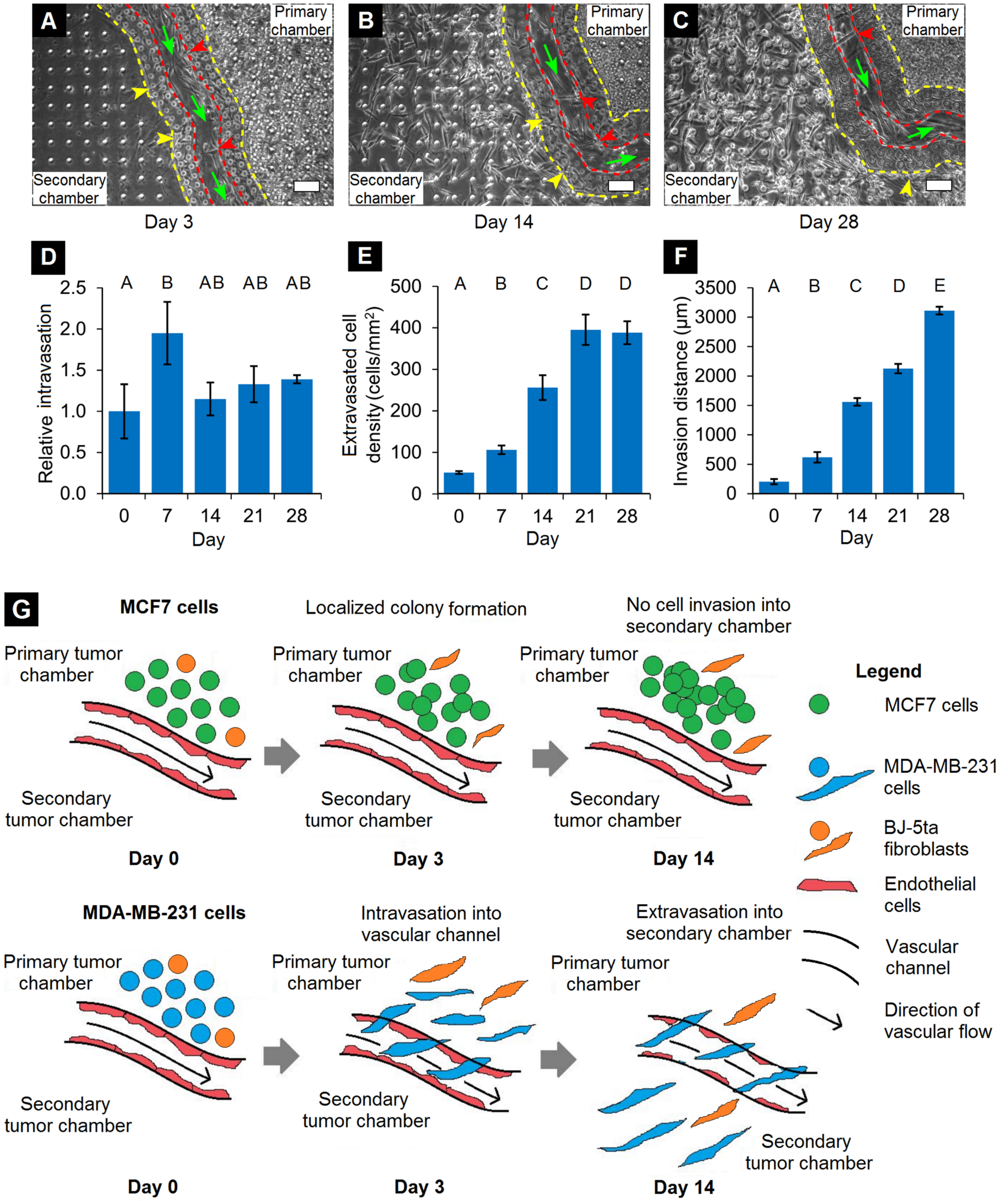

Supplementary Figure S8. Intravasation and extravasation of MDA-MB-231 cells in tumor-mimetic chips. (A) MDA-MB-231 cells (co-encapsulated with fibroblasts) intravasated from the primary tumor chamber into the adjacent vascular channels (red dotted lines) by day 3 (indicated by red arrowheads) and further extravasated into the secondary tumor chamber (indicated by yellow arrowheads). Yellow dotted lines represent edges of the vascular gap separating the tumor chambers from the vascular channel. Green arrows indicate direction of vascular flow. **(B)** Cells

gradually invaded into the secondary chamber through day 14 and **(C)** day 28, with a marked increase in cell density in the secondary tumor chamber over time (Scale bar = 100 μm). **(D)** Relative rate of intravasation into the vascular channel, **(E)** increase of extravasated cell density in the secondary tumor chamber and **(F)** increase in migration distance of the invasive cell front in long-term culture. Groups having different letters have significantly different means ($p < 0.05$); n = 3 independent chips per time point, error bars represent standard deviation. **(G)** Schematic of the comparative tendency of invasion and migration of the two cancer cell lines. MCF7 (co-encapsulated with fibroblasts) remain locally confined in the primary tumor chamber over time, but MDA-MB-231 (co-encapsulated with fibroblasts) cells intravasate into the adjoining vascular channels and extravasate into the secondary tumor chamber over time.

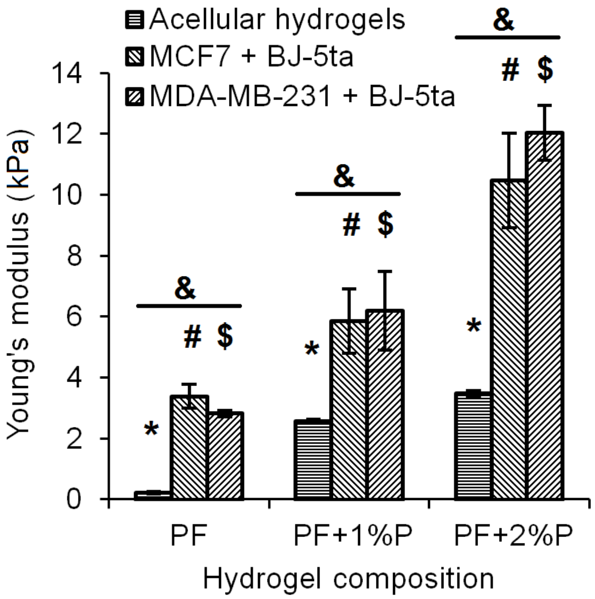

Supplementary Figure S9. Stiffness quantification of tissue-engineered cancer-fibroblast co-cultures within PEG-fibrinogen based hydrogels. Acellular or cell-laden PEG-fibrinogen hydrogels of varying compositions demonstrated increasing Young's moduli with increasing PEGDA concentration. Measurements obtained after 7 days of culture. PF, PF+1%P and PF+2%P denotes PEG-fibrinogen, PEG-fibrinogen with additional 1% w/v PEGDA and PEG-fibrinogen with additional 2% w/v PEGDA respectively. (*, # and $ represent significant difference between acellular hydrogel groups, MCF7 cell-laden hydrogel groups and MDA-MB-231 cell-laden hydrogel groups respectively, $p<0.05$; & represents significant difference between acellular and cellular hydrogel constructs, $p<0.05$, n = 3 independent hydrogels per condition, error bars represent standard deviation).

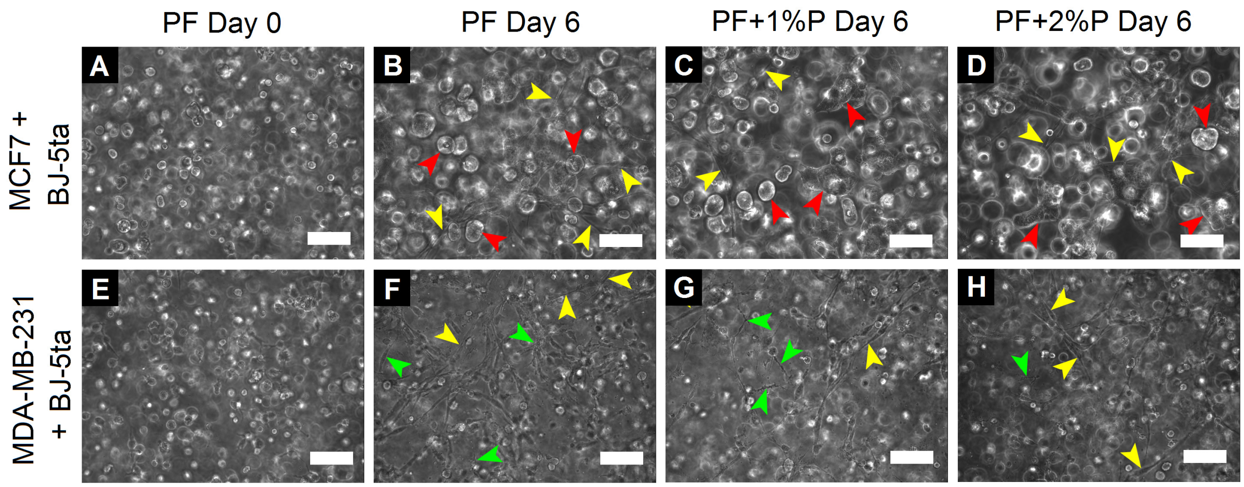

Supplementary Figure S10. Morphological progression of cancer cells and fibroblasts co-cultured within PEG-fibrinogen based hydrogels. (A-D) MCF7 cells and **(E-H)** MDA-MB-231 cells co-encapsulated with BJ-5ta fibroblasts (yellow arrowheads) within PEG-fibrinogen hydrogels of increasing PEGDA concentrations showed morphological changes over time. MCF7 cells formed distinct local colonies (red arrowheads) which appeared more numerous in the PF hydrogels as compared to the PF+1%P or PF+2%P hydrogels. MDA-MB-231 cells appeared more elongated and spread out in the PF hydrogels as compared to the PF+1%P or PF+2%P hydrogels. Scale bar = 100 μm.

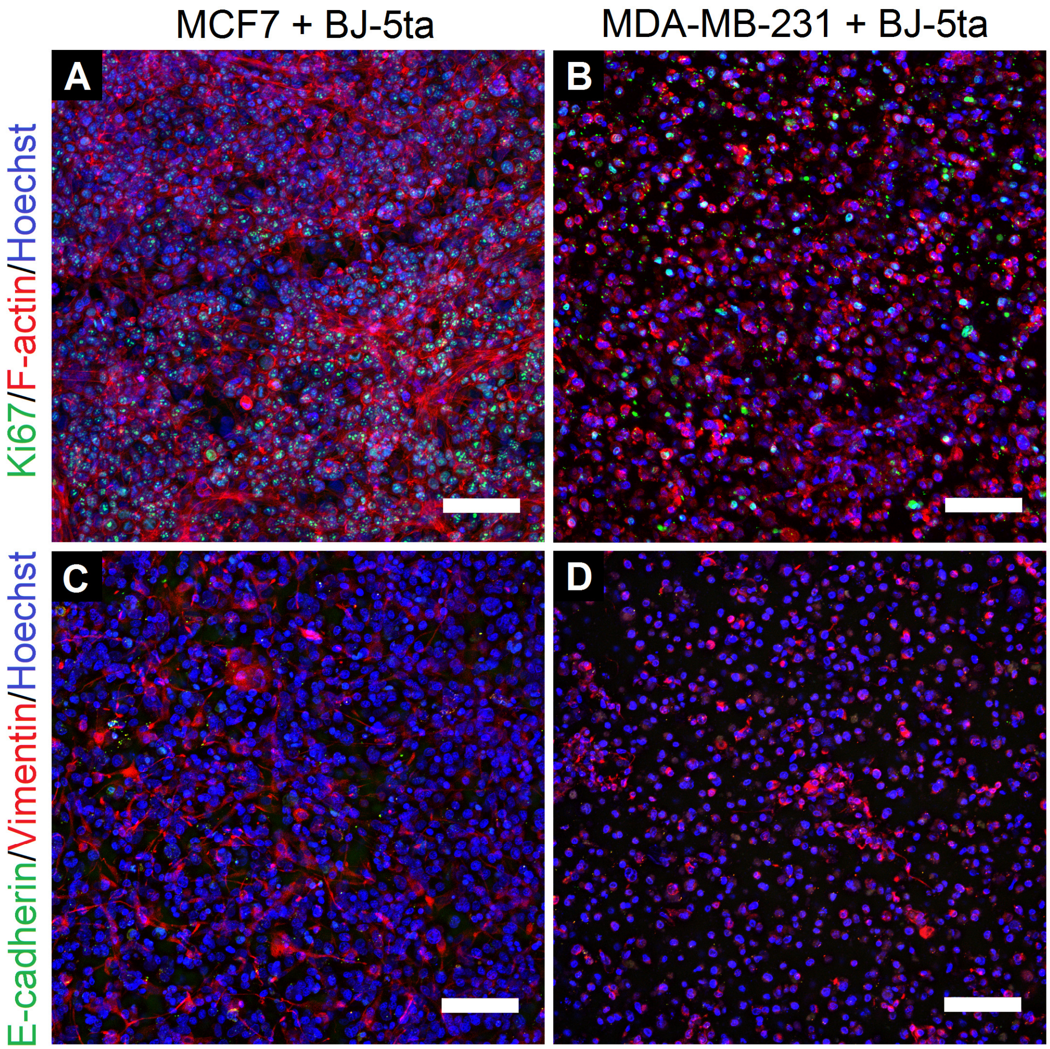

Supplementary Figure S11. Cellular expression of cancer cells and fibroblasts co-cultured within PEG-fibrinogen based hydrogels. Z-stack projections of **(A)** MCF7 cells and **(B)** MDA-MB-231 cells co-encapsulated with BJ-5ta fibroblasts showed a high degree of proliferation and cell spreading (Ki67, green; F-actin, red; nuclei, blue). **(C)** Vimentin was prominently expressed by fibroblasts in co-culture with MCF7 cells and **(D)** MDA-MB-231 cells, but expression of E-Cadherin was relatively low in both cases. Scale bar = 100 μm.

Supplementary Methods:

Analysis of endothelial cell morphology: Fluorescence images of human breast tumor associated endothelial cells (hBTECs) obtained via confocal microscopy were analyzed via ImageJ software (NIH, Version 1.51) to extract morphological characteristics of the cells seeded within the microfluidic channels of the tumor-mimetic chips. The outline of each cell was manually traced based on the CD31 staining (green channel) in the fluorescent images and the cell area, circularity and aspect ratio was directly obtained via automated ImageJ analysis. The geometric diameter of each cell was calculated from the major and minor axes of the analyzed cell according to the following formula[1]:

$$\text{Geometric diameter} = \sqrt{\text{major axis} \times \text{minor axis}} \quad (1)$$

Cell density of endothelial cells was calculated by dividing the number of seeded cells in each channel segment by the surface area of the corresponding segment according to the following formula:

$$\text{Cell density} = \frac{\text{Number of seeded cells in each segment of the channel}}{4 \times \text{Channel width} \times \text{Channel segment length}} \quad (2)$$

Here, channel width = 100 μm for all channel segments.

Circularity and aspect ratio are defined as below:

$$\text{Circularity} = 4\pi \frac{\text{Area}}{\text{Perimeter}^2} \quad (3)$$

$$\text{Aspect ratio} = \frac{\text{Major axis}}{\text{Minor axis}} \quad (4)$$

Elongation length was defined by the maximum distance between two points along the selected cell boundary (Feret's diameter) and was obtained via automated ImageJ analysis of the traced cell boundaries[1].

Alignment of the hBTECs with shear flow was analyzed as described. The relative alignment of each microchannel segment within the fluorescence image was measured based on a horizontal frame of reference. The Feret's angle of each traced cell was automatically obtained via ImageJ and the relative channel alignment angle was subtracted from the Feret's angle to obtain the absolute value of the cellular alignment angle. The frequency of each cellular alignment angle was tabulated, grouped and plotted in MATLAB.

Analysis of cancer-fibroblast co-culture morphology: Cancer cells co-encapsulated with BJ-5ta fibroblasts within the PEG-fibrinogen hydrogel matrix in the microfluidic devices were imaged via phase contrast microscopy every 7 days over the 28 day culture period morphological quantification was conducted via ImageJ software. In the case of MCF7 cells, colonies or clusters

of cells were manually traced and their area, circularity and aspect ratio were automatically extracted via the ImageJ software. The colony diameter was calculated from the major and minor axes of the traced regions according to Formula (1).

MCF7 colony density was defined as:

$$\text{Colony density} = \frac{\text{Number of clusters/colonies in field of view}}{\text{Area of central tumor chamber in the field of view}} \qquad (3)$$

Elongated MCF7 colonies were those distinguished by irregular protrusions in the traced colony boundaries and were characterized as having an aspect ratio > 1.5.

In the case of MDA-MB-231 cells, individual cells were manually traced and cellular area, circularity and aspect ratio was automatically extracted via the ImageJ software. The cellular diameter was calculated in similar manner as above based on Formula (1). The cellular elongation length was considered as the longest distance between two points on the traced cell boundary (Feret's diameter)[1].

Computational modeling of shear flow profiles in microfluidic chips: A general-purpose Computational Fluid Dynamics (CFD) code, CFD-ACE+,[2] based on the Finite Volume Method (FVM) was used to discretize and solve the governing equations. Briefly, a three-dimensional computational mesh was created using CFD-GEOM, the grid generation module of CFD-ACE+. Steady state fluid flow was described by the conservation of fluid mass and momentum (Naviér–Stokes) equations similar to our previous studies[3,4] with inlet flow rates matching the experimental situation. The simulations results were analyzed using CFD-VIEW to obtain the shear maps presented in Figure 4A,B.

Analysis of diffusion gradients within the microfluidic chips: Post-perfusion of TRITC-dextran through the vascular channels of the microfluidic chips, the fluorescence images of the dextran-perfused regions of the central tumor chamber were acquired and analyzed in ImageJ in order to quantify diffusion gradients. The raw images were imported in ImageJ and LUTs corresponding to thermal heatmaps were applied to the images to generate visual gradients of the perfused dextran. Selected perfusion directions were measured for each chip design as denoted by arrows (Fig. 4E, F) and the 'Plot Profile' function of ImageJ was used to obtain quantitative values of the fluorescence intensity along each line. The values were normalized to the fluorescence intensity value of the TRITC-dextran in the surrounding vascular channel.

Analysis of drug-treatment effects in tumor-mimetic chips: Post-drug treatment and live/dead image acquisition of cells within the tumor-mimetic chips, images were analyzed in ImageJ software to evaluate the two parameters: viable cell density and viable tumor area. The number of

viable cells (stained green) were manually counted within each field of view and reported in terms of these two parameters.

Viable cell density is defined as the relative percentage of viable cells present within the field of view. The number of live cells counted in each field of view were compared to the control condition and expressed as a percentage to obtain this value.

Viable tumor area is defined as the relative area in the field of view occupied by the viable cells. The overall area occupied by the viable cells within the central tumor chamber was assessed, compared to the control condition and reported as a percentage to obtain this value.

For endothelial cells in the vascular channels and for cancer cells within static 3D cultures, the viable cell density was estimated in a similar manner as described above.

Analysis of MDA-MB-231 cell dynamics within tumor-mimetic chips: MDA-MB-231 cells co-encapsulated with fibroblasts in the PF hydrogel matrix within the tumor-mimetic chips were observed over 28 days in dynamic culture. MDA-MB-231 cells were observed to intravasate from the primary tumor chamber into the adjoining vascular channels as was quantified as the relative intravasation. The number of cancer cells present in the vascular channels at a specific time point was manually counted from phase contrast images and normalized to the number of cells at the initial time point to obtain the relative intravasation value.

The intravasated cancer cells further invaded into the adjacent secondary tumor chamber through long-term culture and the extravasated cell density was quantified by manually counting the number of cancer cells in the secondary tumor chamber and dividing it by the area of the chamber in the field of view. The invasion distance was calculated by measuring the distance covered by the invading cell front after crossing over into the secondary chamber.

References:

1. Pradhan, S., Hassani, I., Seeto, W. J. & Lipke, E. A. PEG-fibrinogen hydrogels for three-dimensional breast cancer cell culture. *J. Biomed. Mater. Res. A* **105**, 236-252, (2017).
2. Jiang, Y., Przekwas, A.J. Implicit, pressure-based incompressible Navier-Stokes equations solver for unstructured meshes., 32nd Aerospace Sciences Meeting and Exhibit, AIAA , Reno, NV; United States; (1994).
3. Prabhakarpandian, B. et al. Synthetic microvascular networks for quantitative analysis of particle adhesion. Biomed. Microdevices **10**, 585-595, (2008)
4. Lamberti G., Tang Y., Prabhakarpandian B., Wang Y., Pant K., Kiani M.F., Wang B. Adhesive interaction of functionalized particles and endothelium in idealized microvascular networks. Microvasc. Res.; **89**:107-114 (2013).
